# Recovery of Metals from the “Black Mass” of Waste Portable Li-Ion Batteries with Choline Chloride-Based Deep Eutectic Solvents and Bi-Functional Ionic Liquids by Solvent Extraction

**DOI:** 10.3390/molecules29133142

**Published:** 2024-07-02

**Authors:** Urszula Domańska, Anna Wiśniewska, Zbigniew Dąbrowski, Dorota Kolasa, Kamil Wróbel, Jakub Lach

**Affiliations:** Łukasiewicz Research Network—Industrial Chemistry Institute, Rydygiera 8, 01-793 Warsaw, Poland; anna.wisniewska@ichp.lukasiewicz.gov.pl (A.W.); zbigniew.dabrowski@ichp.lukasiewicz.gov.pl (Z.D.); dorota.kolasa@ichp.lukasiewicz.gov.pl (D.K.); kamil.wrobel@ichp.lukasiewicz.gov.pl (K.W.); jakub.lach@ichp.lukasiewicz.gov.pl (J.L.)

**Keywords:** metal extraction from black mass, DESs, bi-functional ionic liquids, extraction efficiency

## Abstract

Lithium-ion portable batteries (LiPBs) contain valuable elements such as cobalt (Co), nickel (Ni), copper (Cu), lithium (Li) and manganese (Mn), which can be recovered through solid–liquid extraction using choline chloride-based Deep Eutectic Solvents (DESs) and bi-functional ionic liquids (ILs). This study was carried out to investigate the extraction of metals from solid powder, black mass (BM), obtained from LiPBs, with various solvents used: six choline chloride-based DESs in combination with organic acids: lactic acid (1:2, DES 1), malonic acid (1:1, DES 2), succinic acid (1:1, DES 3), glutaric acid (1:1, DES 4) and citric acid (1:1, DES 5 and 2:1, DES 6). Various additives, such as didecyldimethylammonium chloride (DDACl) surfactant, hydrogen peroxide (H_2_O_2_), trichloroisocyanuric acid (TCCA), sodium dichloroisocyanurate (NaDCC), pentapotassium bis(peroxymonosulphate) bis(sulphate) (PHM), (glycine + H_2_O_2_) or (glutaric acid + H_2_O_2_) were used. The best efficiency of metal extraction was obtained with the mixture of {DES 2 + 15 g of glycine + H_2_O_2_} in two-stage extraction at pH = 3, *T* = 333 K, 2 h. In order to obtain better extraction efficiency towards Co, Ni, Li and Mn (100%) and for Cu (75%), the addition of glycine was used. The obtained extraction results using choline chloride-based DESs were compared with those obtained with three bi-functional ILs: didecyldimethylammonium bis(2,4,4-trimethylpentyl) phosphinate, [N_10,10,1,1_][Cyanex272], didecyldimethylammonium bis(2-ethylhexyl) phosphate, [N_10,10,1,1_][D2EHPA], and trihexyltetradecylphosphonium bis(2,4,4-trimethylpentyl) phosphinate, [P_6,6,6,14_][Cyanex272]/toluene. The results of the extraction of all metal ions with these bi-functional ILs were only at the level of 35–50 wt%. The content of metal ions in aqueous and stripped organic solutions was determined by ICP-OES. In this work, we propose an alternative and highly efficient concept for the extraction of valuable metals from BM of LiPBs using DESs and ILs at low temperatures instead of acid leaching at high temperatures.

## 1. Introduction

In recent years, the number of lithium-ion portable batteries (LiPBs) has increased rapidly because they are widely used as electronic tools due to their superior electrical performance. LiPBs contain metals such as Li, Co, Ni, Mn, Cu, Zn and Al, as well as polymers, ceramics and other substances [1,2,3]. In a typical Li-ion battery, the positive electrode material is an intercalated lithium compound, e.g., LiCoO_2_, LiMn_2_O_4_, lithium nickel manganese cobalt oxides LiNi_x_Mn_y_Co_1−x−y_O_2_ (abbreviated NMC, Li-NMC, LNMC or NCM) or LiFePO_4_, while Cu, Al and Zn are present in the electrode current collectors and in the structural elements of the cells [4]. As the amount of LiPBs used increases, waste containing hazardous substances is generated, which is harmful to human life and health and to the environment (Ni and its compounds, together with Cd, Hg, Pb and their compounds, are included in the group of so-called “priority substances” presenting a significant risk to or via the aquatic environment [5,6]). The regulations on batteries and accumulators in force in the European Union (new Regulation [7], replacing Directive [8] after the transitional period) recommend the development of new, environmentally friendly technologies for the processing and recycling of waste batteries and accumulators [9]. Therefore, the aims are to recover metals from solid powder, black mass (BM), with efficient and green methods using solvents such as Deep Eutectic Solvents (DESs) and ionic liquids (ILs). DESs are environmentally friendly solvents used to replace traditional solvents for a given application [10,11]. DESs are attractive solvents due to their intriguing physicochemical properties, which are a function of the nature of the hydrogen bond acceptor (HBA) and donor (HBD) and their molar ratio [12]. Most DESs are based on choline chloride (ChCl) and organic acids or ethylene glycol, betaine, glycerol and others in different molar ratios [12]. Extraction proceeds from the solid phase to the liquid phase, in which the separation of metals from each other must be realized via successive solvent extractions [13,14]. Given the rapid growth of the market share of lithium-ion batteries (LiBs), now used in electric and hybrid vehicles, laptops, cellphones and energy storage facilities, recycling of Co, Li, Ni, Mn, Al and Cu is very important (these metals are on the list of critical raw materials [15]). The prospect of global production of LiBs will probably amount to 93.1 billion US dollars in 2025. The production of LiBs is expected to gradually increase [16].

A new solvent extraction technology has been proposed by many authors, using different extraction procedures, different extraction solvents with different additives and at different pHs, temperatures and times to recover valuable elements from BM [1,2,3,17]. Organic acids are widely used instead of inorganic acids and has been described in important reviews [13,14]. The use of DESs and ILs in the metal recycling procedure based on direct solid–liquid leaching decreases the costs of the extraction versus acid leaching (not green); at high temperatures (360–770 K), times of extraction, greenhouse gasses; and with huge amounts of water. However, the primary challenge and opportunity for DESs and ILs is to achieve high selectivity compared to inorganic acids, benchmark extractants and diluents. The extraction of Co, Li and Ni from BM of waste LiBs using ILs, DESs and organophosphorous-based acids has been well described in our previous work [18]. Very promising results were obtained with DES (choline chloride, [N_2OH,1,1,1_][Cl]: lactic acid, 1:2), giving an extraction efficiency of Co 88.5%, Li 86.7% and Ni 84.5% at pH = 2.5, and DES (choline chloride, [N_2OH,1,1,1_][Cl]:phenylacetic acid, 1:2), giving a Co extraction efficiency of 98.7% and Li 100% at O:A = 1:1 [18]. Unfortunately, the BM of spent LiPBs versus LiBs contains different amounts of metals, especially much less Co and Li, which is confirmed in this work. The proposed process, which could recover Co, Ni, Li, Cu and Mn simultaneously from the solid to the liquid phase, is believed to be economical and environmentally friendly. Therefore, we grant attention to ILs, where the hydrocarbon chains on the cation are appreciably larger to allow them to be effective surface-active compounds. The most popular ILs are with ammonium or phosphonium cations and carboxylic or thiocyanate anions, especially for extraction from the aqueous phase [19]. The extraction from the solid phase is usually performed using DESs or ILs (tributylmethylammonium chloride [N_4,4,4,1_][Cl], trihexyl tetradecylphosphonium chloride, [P_6,6,6,14_][Cl]) with the addition of various oxidizing additives such as hydrogen peroxide, H_2_O_2_, trichloroisocyanuric acid (TCCA), (glycine + H_2_O_2_) or (glutaric acid + H_2_O_2_) [20,21,22]. A recent study [23] has proposed the extraction of Co(II), Li(I) and Ni(II) from the anodic and cathodic powder of laptops after leaching with 1.5 M H_2_SO_4_ with the addition of 30% H_2_O_2_ or/and glutaric acid at the temperature *T* = 328 K or 363 K at a solid to liquid phase ratio of 1:10 [23].

Our recent studies have shown the extraction of elements using ILs, DESs, bis(2,4,4-trimethylpentyl)phosphinic acid and Cyanex 272 from waste printed circuit boards (WPCBs) after thermal pre-treatment and leaching with various acids [24]. ILs, the Aqueous Biphasic System (ABS) method and DESs were used to extract metals from leachates and from the solid phase to the extent of 20–30 wt% of Ag, Cu and other metals [24]. 

An excellent overview of metals extraction using ILs and DESs with the addition of various substances, as well as using organophosphorous-based acids, is presented in the works of Liu et al. [25] and Barrueto et al. [26]. Organophosphorous-based acids such as Cyanex 272, bis(2,4,4,-trimethylpentyl)dithiophosphinic acid (Cyanex-301), di-2-ethylhexyl phosphoric acid (D2EHPA) and 2-ethylhexyl phosphonic acid-mono-2-ethylhexyl ester (PC88A) are known as effective extractants for the separation of cobalt and nickel, showing higher selectivity of cobalt over nickel. The separation of Co(II), Ni(II) and Li(I) can be achieved using oxalic acid salts, Mn(II) using D2EHPA, Ni(II) using C_4_H_8_N_2_O_2_ and Co(II) using (NH_4_)_2_C_2_O_4_ [27]. The extraction of Co using PC-88A at pH = 6.5 has been proposed [28]. The precipitation of Li(I) from an aqueous solution containing 5 g /dm^3^ of Li^+^ can be achieved using Na_3_PO_4_, Na_2_CO_3_ and K_2_HPO_4_ [29]. The possibility of extracting 95% Li^+^ from LiBs leachate using a new IL (carboxymethyl trimethylammonium bis(trifluoromethylsulfonyl)imide), [N_CM,1,1,1_][NTf_2_] with O/A ratio = 2:1 in time of 20 min, at pH = 3 and also extraction of other metals, such as Ni, Co, Mn, at pH > 3 and temperature *T* = 298 K, has also been proposed [30]. The extraction of Co (98.23%) from cell phone powdered LiBs using [N_8,8,8,1_][Cl] (Aliquat 336) at pH = 1 and O/A =1.5 is recommended in [31]. The extraction of Co and Ni from the LIB’s cathode material, LiCoO_2,_ was proposed using DESs [32]. High Co extraction efficiency (99.6 wt%) was obtained with DES (choline chloride + citric acid, 1:1 or 2:1) [32]. Efficient extraction of Co from the LIB’s cathode material, LiCoO_2_, was also proposed using 0.5 M glycine (chelating agent) and 0.02 M ascorbic acid (reductant) at *T* = 353 K for 1 h after the carboxylation process at *T* = 973 K for 2 h [33]. Literature data on the physicochemical properties of some DESs and their extraction properties are described in [34].

The use of a bi-functional IL synthesized from Aliquat 336 and D2EHPA, cationic and anionic extractants, increased the extraction of Mo and V from the spent petroleum catalysts by more than 30% [35]. Good results in the selective and effective leaching of vanadium and molibdenium from the spent petroleum catalysts were obtained with bi-functional IL composed of Alamine 336 (*N*,*N*-dioctyl-1-octanamine) and ionic liquid Ali-D2 in the presence of oxidizing agent, H_2_O_2_ [36].

In the solvometallurgy process, a combination of solvent extraction and gradient chemical precipitation is usually used to separate and recover transition metals from the leachate. Since metal ions in solutions, such as Co(II), Ni(II), Cu(II), Li(I) and Mn(II), are similar in nature, a lot of methods and solvents have been proposed in recent years to separate them from each other [37,38,39,40,41,42]. However, this recycling process requires the use of additional energy and chemical reagents, and it will be the next step in our recycling experiments. 

In this paper, the extraction performance of Co, Ni, Li, Cu and Mn from LIPBs’ BM has been optimized using different DESs: (1) {choline chloride + lactic acid, 1:2}, (2) {choline chloride + malonic acid, 1:1}, (3) {choline chloride + succinic acid, 1:1}, (4) {choline chloride + glutaric acid, 1:1}, (5,6) {choline chloride + citric acid, 1:1 and 2:1} and bi-functional ILs: didecyldimethylammonium bis(2,4,4-trimethylpentyl) phosphinate, [N_10,10,1,1_][Cyanex272], didecyldimethylammonium bis(2-ethylhexyl) phosphate, [N_10,10,1,1_][D2EHPA], trihexyltetradecylphosphonium bis(2,4,4-trimethylpentyl) phosphinate, [P_6,6,6,14_][Cyanex272]/toluene (an aromatic diluent). Various additives have been used, such as a didecyldimethylammonium chloride (DDACl) surfactant, hydrogen peroxide (H_2_O_2_), trichloroisocyanuric acid (TCCA), sodium dichloroisocyanurate (NaDCC), pentapotassium bis(peroxymonosulphate) bis(sulphate) (PHM), (glycine + H_2_O_2_), or (glutaric acid + H_2_O_2_). 

These processes mainly consisted of solid–liquid extraction at different process parameters such as pH, temperature, time and solid to liquid phase ratio. The metal ions concentration in aqueous and stripped organic solutions was determined by the ICP-OES method.

## 2. Results and Discussion

### 2.1. Solid BM Composition

**Figure 1:**
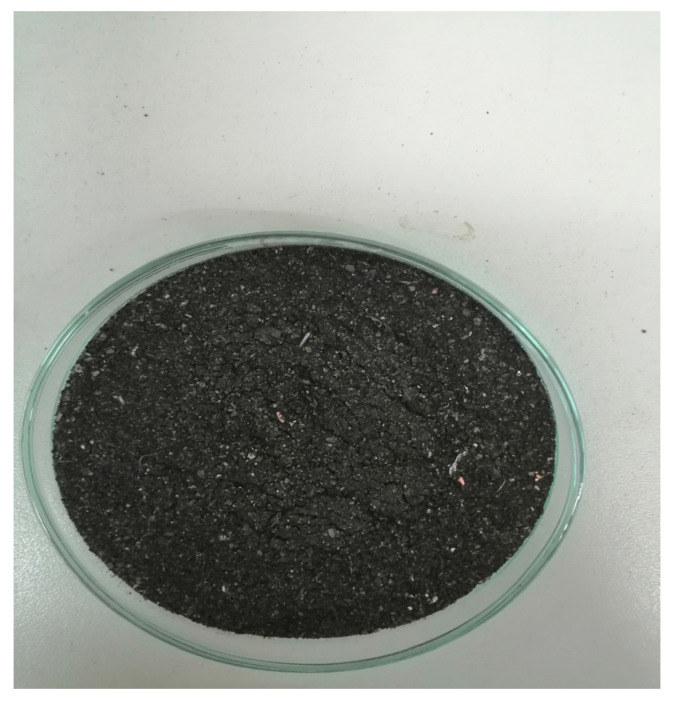
Solid LiPBs’ BM sample before powdering in a mortar.

**Figure 2:**
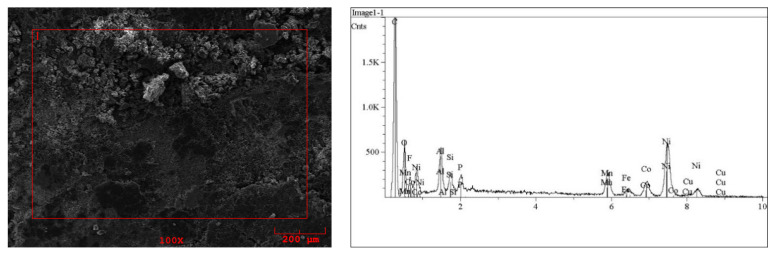
SEM image of the solid LiPBs’ BM sample and EDS spectrum of the micro-area marked in the image.

The content of valuable elements (Co, Ni, Cu, Mn, Fe, Al, Zn and Li) in spent LiNi_x_Mn_y_Co_1−x−y_O_2_ powder is given in Table 1. The material was manually shredded and ground in a mortar into small particles with a diameter of 1.2 mm (see Figure 1). The results of the analysis of the metal content in the starting BM material provided by the microwave digestion method/FAAS and FAES techniques are presented as the EDS spectra concerning only the micro area of the BM surface in Figure 2 and in Figures S1–S3 in the Supplementary Material (SMs).

The starting BM contained the following metals: cobalt (Co), nickel (Ni), copper (Cu), manganese (Mn), iron (Fe), aluminum (Al), zinc (Zn) and lithium (Li). The metal content was about 0.1–8.6 wt%. The BM of LiPBs contained significantly less Co (2.95 wt%) and Li (2.2 wt %) compared to the previously studied BM of LiBs (Co 26.0 wt%, Li 4.0 wt%) [20]. On the other hand, the BM of LiPBs contained more Cu (4.0 wt%) compared to the BM of LiBs (2.8 wt%) [20]. This result provides a proof of concept for the use of modern-green solvents as liquids to enable the recycling of smaller amounts of Co. It also requires better technique and better extraction parameters, especially in the next step of Co-Ni separation.

### 2.2. Extraction

The metal extraction efficiency (*E*) was calculated according to the following formula:

*E* (wt%) = 100 × (g_E,A_ + g_E,O_)/g_0_ (1)

where g_0_ (g) is the initial metal content in the solid material and g_E_ (g) is the content of metal ion in the aqueous phase (A) and the organic phase (O) after extraction.

#### 2.2.1. Extraction with DESs

In general, most DESs possess a relatively high viscosity (>100 cP) at room temperature, which significantly limits their application for extraction. Their high viscosity reduces the mass transfer rate between the sample and the extraction phase, owing to the formation of extensive hydrogen bond networks between the hydrogen bond acceptor, HBA (choline chloride), and the hydrogen bond donor, HBD, (organic acid) component. There are two methods to reduce the viscosity: increasing the temperature or adding the diluent. The addition of water to DESs significantly reduces the viscosity of DESs as a result of the gradually weakened hydrogen bonding interaction between the DES components. The interaction between HBA and HBD is weakened or even disappears when the water content is above 50% (*v*/*v*). The hydrogen bond interactions between the HBA and HBD are reduced, and, at this stage, the DES loses its unique eutectic properties and exists like a liquid with individual HBA and HBD components [43]. 

The results of the extraction with DES 1 and DES 2 are summarized in Table 2 and Table 3, respectively. The extraction efficiency (*E*) obtained with these DESs is appreciably much higher than those using other DESs or bi-functional ILs. The extraction efficiency with DES 1 exhibited effective but simultaneous extraction of all metals, including Co, Ni, Li, Cu and Mn at low temperatures, *T* = 333 K and at a short time of 2 h, at pH = 3. The results depend on oxidizing additives at constant temperature, time and pH. The best results for DES 1 were observed for TCCA, *E*_Co_ = 48 wt%, *E*_Ni_ = 57 wt%, *E*_Li_ = 56 wt% and *E*_Mn_ = 60 wt%. Similar results were also obtained for (glycine + H_2_O_2_): *E*_Co_ = 52 wt%, *E*_Ni_ = 53 wt%, *E*_Li_ = 54 wt% and *E*_Mn_ = 62 wt%. A high extraction efficiency was observed for copper: *E*_Cu_ = 95 wt% for (DES 1 + H_2_O_2_), *E*_Cu_ = 97 wt% for (DES 1 + PHM), *E*_Cu_ = 70 wt% for (DES 1 + glycine + H_2_O_2_) and *E*_Cu_ = 82 wt% for (DES 1 + glutaric acid + H_2_O_2_). An interesting fact that is worth noting when viewing Table 2 is that the best extraction efficiency of Co is at the level of 48–52 wt% or for Ni at 53–57 wt% when using (DES 1 + TCCA) or (DES 1 + glycine + H_2_O_2_). However, the results expected by the industry should exceed an extraction efficiency higher than 75%.

The extraction efficiency with DES 2 also showed very good extraction of all metals at the same temperature, time and pH. The best results were observed for (DES 2 + glycine + H_2_O_2_): *E*_Co_ = 60 wt%, *E*_Ni_ = 66 wt%, *E*_Li_ = 67 wt%, *E*_Cu_ = 75 wt% and *E*_Mn_ = 65 wt%. Similar results were also obtained for (DES 2 + TCCA): *E*_Co_ = 59 wt%, *E*_Ni_ = 50 wt%, *E*_Li_ = 65 wt%, *E*_Cu_ = 86 wt% and *E*_Mn_ = 69 wt%, and for (DES 2 + NaDCC × 2H_2_O): *E*_Co_ = 57 wt%, *E*_Ni_ = 56 wt%, *E*_Li_ = 66 wt%, *E*_Cu_ = 37 wt% and *E*_Mn_ = 62 wt%. The system (DES 2 + H_2_O_2_) revealed only high extraction of Li, *E*_Li_ = 76 wt%. The best extraction efficiency for Co is at the level of 60 wt% or for Ni 50–66 wt% using (DES 2 + TCCA) or (DES 2 + NaDCC × 2H_2_O) or (DES 2 + glycine + H_2_O_2_) (see Table 3). 

These results confirmed that DES 1 or DES 2 is able to extract metals from the solid phase with an extraction efficiency higher than 75% only for Cu and Li in the presence of H_2_O_2_, TCCA, PHM and the DDACl surfactant. 

The extraction efficiency with DES 3 shows lower metal extraction effects. The best results were observed for (DES 3 + TCCA): *E*_Co_ = 54 wt%, *E*_Ni_ = 44 wt%, *E*_Li_ = 73 wt%, *E*_Cu_ = 94 wt% and *E*_Mn_ = 73 wt%. Similar results were also obtained for (DES 2 + TCCA): *E*_Co_ = 59 wt%, *E*_Ni_ = 50 wt%, *E*_Li_ = 65 wt%, *E*_Cu_ = 86 wt% and *E*_Mn_ = 69 wt%. Slightly worse results were observed for (DES 3 + NaDCC × 2H_2_O): *E*_Li_ = 60 wt% and *E*_Cu_ = 48 wt%, and for the rest of metals, the extraction efficiency was <20 wt%. The system (DES 3 + H_2_O_2_) revealed considerable extraction only in the case of Li, *E*_Li_ = 48 wt% and of Mn, *E*_Mn_ = 42 wt%. Unfortunately, the extraction efficiency using (DES 3 + glycine + H_2_O_2_) was not attractive and only lithium extraction was at the level of *E*_Li_ = 55 wt%. Low extraction efficiency was also observed for (DES 3 + PHM) for all metals except lithium and manganese, *E*_Li_ = 64 wt% and *E*_Mn_ = 55 wt%. The best extraction efficiency for Co was 53 wt% using (DES 3 + TCCA) and for Ni 44–49 wt% using (DES 3 + TCCA) or (DES 3 + glycine + H_2_O_2_) or (DES 3 + glutaric acid + H_2_O_2_) (see Table 4). As in the case of DESs 1 and 2, extraction efficiencies above 75% were only observed for Cu in the presence of TCCA and the DDACl surfactant.

The DES 4 with the addition of TCCA shows good recovery of Co, Ni, Li, Cu and Mn: *E*_Co_ = 51 wt%, *E*_Ni_ = 50 wt%, *E*_Li_ = 60 wt%, *E*_Cu_ = 87 wt% and *E*_M n_= 65 wt%. Much smaller extraction effects were observed for DES 4 with the addition of H_2_O_2_. All extraction efficiencies were lower than 50 wt%. Only affinity for Mn was observed, *E*_Mn_ = 53 wt%. The extraction of metals was very low using (DES 4 + NaDCC × 2H_2_O), and only the extraction of Li was >50 wt%, *E*_Li_ = 51 wt%, and for the rest of metals the extraction efficiency was ≤20–30 wt%. The aqueous phase and NaDCC × 2H_2_O do not interact with the cationic functional group. For the same reasons, not very attractive selectivity was also observed for (DES 4 + PHM): *E*_Li_ = 65 wt% and *E*_Mn_ = 61 wt% in one aqueous phase. The extraction efficiency using (DES 4 + glycine + H_2_O_2_) was not very significant, except *E*_Cu_= 76 wt%, *E*_Li_ = 49 wt% and *E*_Mn_ = 48 wt%; for Ni, it was close to 40%. This can be attributed to the physical imprint that does not fit the glycine molecules. The addition of the glutaric acid revealed the extraction efficiency for all metal ions < 50 wt%. The best extraction efficiency for Co was 51 wt% when using (DES 4 + TCCA), and for Ni it was 50 wt% or 43 wt% using (DES 4 + TCCA) or (DES 4 + glutaric acid + H_2_O_2_) (see Table 5). 

Based on the above extraction behaviours of DES 4 and the earlier discussed DESs 1–3, it appears that the type of additive is an important parameter in the extraction of selected metal species in the DESs leachate. Acceptable extraction efficiency was observed only for DES 4 + TCCA or (glycine + H_2_O_2_) for Cu. 

The DES 5 (1:1) with the addition of H_2_O_2_ shows good recovery of Co, Ni, Li, and Mn: *E*_Co_ = 50 wt%, *E*_N i_= 51 wt%, *E*_Li_ = 62 wt% and *E*_Mn_ = 61 wt%. Similar results were obtained with the addition of TCCA. The best results were observed for Cu, *E*_Cu_ = 84 wt% and *E*_Cu_ = 88 wt% for both extractants, respectively. Metal extraction was very effective for (DES 5 (1:1) + NaDCC × 2H_2_O) only for Cu, *E*_Cu_ = 94 wt%. For the rest of the metals, the extraction efficiency was 48–66 wt%. A high extraction efficiency for Li, Cu and Mn was observed for (DES 5 + PHM): *E*_Li_ = 88 wt%, *E*_Cu_ = 87 wt% and *E*_Mn_ = 74 wt%. The extraction efficiency using (DES 5 + glycine + H_2_O_2_) was not very significant, *E*_Cu_ = 85 wt% and *E*_Li_ = 61 wt%, and for the rest of metals was approximately 40–50 wt%. The addition of glutaric acid showed an extraction efficiency for Co, Ni and Mn of <50 wt%, for Li of *E*_Li_ = 53 wt%, and for Cu of *E*_Cu_ = 69 wt% (see Table 6). All DESs 1–5 with all oxidizing additives reviled that the extraction efficiency for Co and Ni was not higher than 50%, and only Cu and Li were extracted with a higher extraction efficiency.

The DES 6 (2:1) with the addition of H_2_O_2_ shows a good recovery of metals: *E*_Co_ = 52 wt%, *E*_Ni_ = 59 wt%, *E*_Li_ = 51 wt%, *E*_Cu_ = 68 wt% and *E*_Mn_ = 62 wt%. Similar results were obtained with the addition of TCCA (except Ni), and the best results were observed for Mn, *E*_Mn_ = 96 wt%. Metal extraction was very effective for (DES 6 (2:1) + NaDCC × 2H_2_O) only for Cu, *E*_Cu_ = 65 wt%, and for Mn, *E*_Mn_ = 68 wt%. For the rest of the metals, the extraction efficiency was 11–42 wt%. Attractive selectivity for Li, Cu and Mn was observed for (DES 6 + PHM): *E*_Li_ = 62 wt%, *E*_Cu_ = 66 wt% and *E*_Mn_ = 83 wt%. The extraction efficiency using (DES 6 + glycine + H_2_O_2_) was very significant for Cu, *E*_Cu_= 71 wt%, and for Mn, *E*_Mn_= 84 wt%; for the rest of metals, it was 20–57 wt%. The addition of glutaric acid revealed the extraction efficiency for Co, Ni and Li at the level of approximately 50–57 wt%, for Cu of *E*_Cu_= 68 wt% and for Mn of *E*_Mn_= 80 wt% (see Table 7). The results presented in Table 7 indicate that only Mn can be extracted at an economical level higher than 75%.

**Figure 3:**
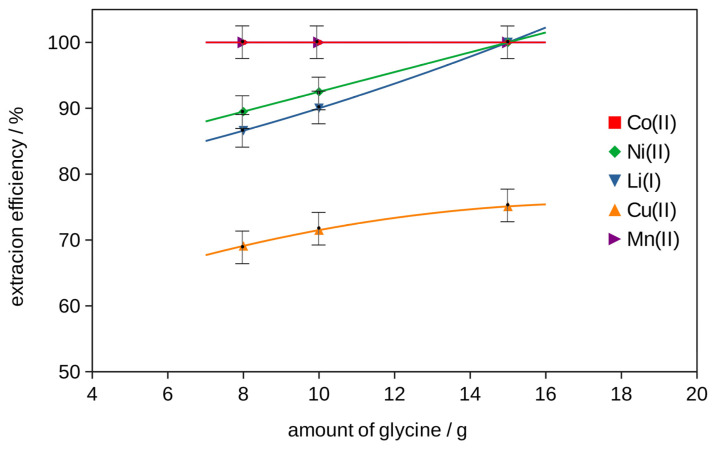
The effect of the amount of glycine on the efficiency of metal ion extraction at temperature *T* = 333 K, pH = 3, and time 2 h, with two-stage extraction.

**Figure 4:**
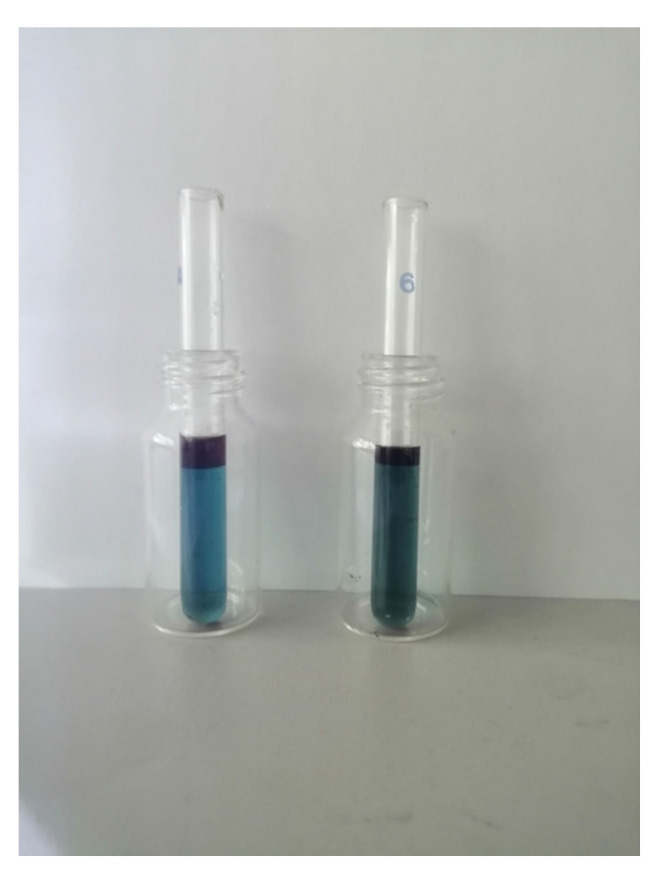
Products of extraction at temperature *T* = 333 K, pH = 3, time 2 h, and two-stage extraction with glycine addition (**left**, 8 g of glycine, **right**, 15 g of glycine).

The best extraction efficiency for all metals was obtained with (DES 2 + glycine + H_2_O_2_), so two-stage extraction was investigated to increase the effect with different amounts of glycine. The results obtained at pH = 3, *T* = 333 K, 2 h, shown in Table S1 in SMs and in Figure 3 and Figure 4, exhibited an increase in the extraction efficiency of metals with an increase in the glycine content in the extractant. The majority of metals were extracted in 100 wt% in the system with the addition of 15 g of glycine. Only Cu’s extraction was 75 wt%.

**Figure 5:**
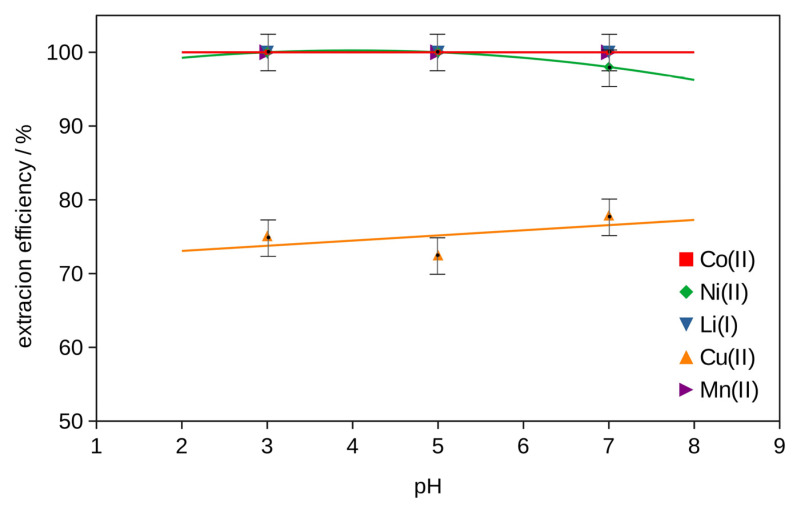
Efficiency of metal ion extraction at *T* = 333 K, 2 h, with 15 g of glycine and two-stage extraction at different pHs.

**Figure 6:**
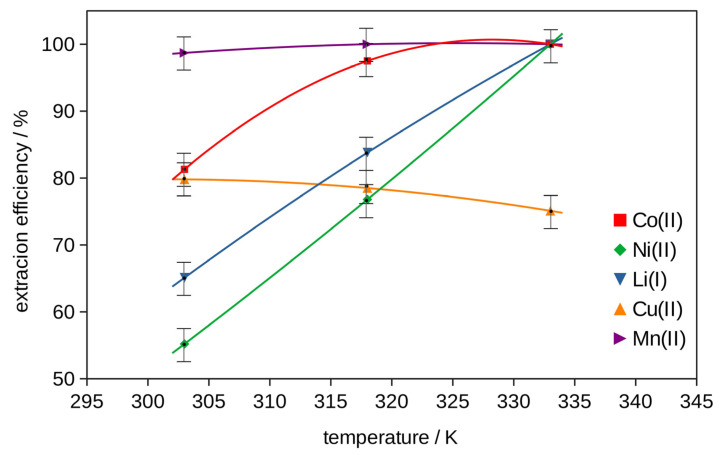
Efficiency of metal ion extraction at pH = 3, 2 h, with 15 g of glycine, and two-stage extraction at different temperatures.

The effect of the pH of the mixture of (DES 2 + 15 g of glycine + H_2_O_2_) was not significant (see Table S2 in SMs and Figure 5). Only the extraction efficiency of lithium and copper increases slightly with the increase in pH. The influence of temperature on the extraction for the same mixture (DES 2 + 15 g of glycine + H_2_O_2_) at pH = 3 is evident. The extraction increases with the increase in temperature from 303 K to 333 K, except for Cu. The best results with an extraction efficiency of 100 wt% were observed for all metals except Cu, 75 wt% (see Table S3 in SMs and Figure 6).

**Figure 7:**
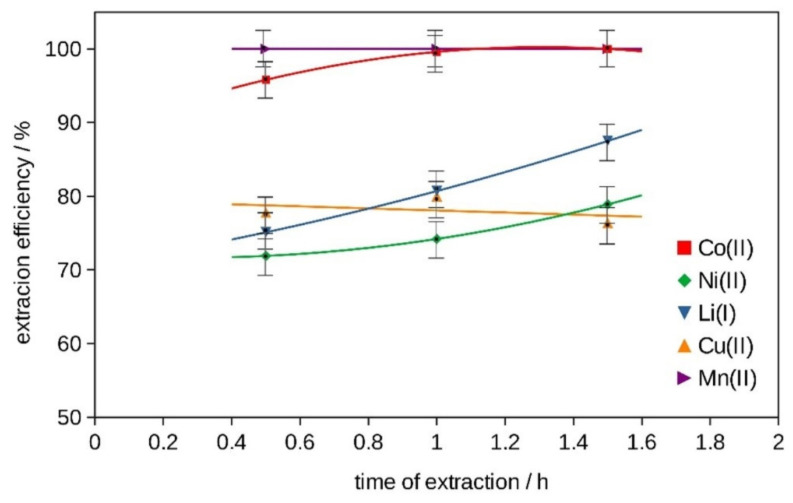
Efficiency of metal ion extraction at *T* = 333 K, pH = 3, with 15 g of glycine and two-stage extraction at different extraction times.

The influence of the time of the two-stage extraction process (*t* = 0.5 h, 1 h, 1.5 h, 2 h) with 15 g of glycine at pH = 3 on the extraction efficiency using the best selected procedure is shown in Table S4 in SMs and in Figure 7. Again, an increase in the extraction efficiency was observed for all metals (100 wt% for Co and Mn except for Cu 75 wt%.

The mechanism of using a composite of ionic liquid (tetrabutylmethylammonium chloride (N_4,4,4,1_Cl) with TCCA for leaching gold from the ore and spent catalyst was presented earlier [20]. During the leaching process, TCCA acted as a metal oxidant and the oxidized metal ions were then captured by IL, which coordinates well with metal ions. A protic solvent such as water, alcohol or acetone induces the formation of cyanuric acid (the product of TCCA reaction). The possible reaction mechanism presented the metallic gold (Au^0^) oxidizing process by TCCA and the resultant [AuCl_3_] then coordinates with Cl^−^ in IL (DES and DDACl in our work) to form [AuCl_4_]^−^. Then, a more stable ion pair is formed with [N_4441_]^+^ through electrostatic attraction, hydrogen bonding and other forces, and finally diffuses into the bulk phase of IL (DES in our work) [20]. Therefore, N_4441_Cl:TCCA:Au = 300:30:1 was assumed as the appropriate mass ratio in that work [20]. It can only be assumed that H_2_O_2_, NaDCC × 2H_2_O and PHM play a similar role. Additionally, DDACl as an IL provides Cl^−^ anions and acts as a surfactant thanks to long aliphatic chains (C_10_).

More than 95% of Co was extracted from spent LIBs using 0.5 M glycine (chelating agent) and 0.02 M ascorbic acid (reductant) at 80 °C for 6 h. UV–vis spectra of the solution confirm the build–up of the Co(III) and Co(II)−glycine complex (λ_max_ ≈ 310 nm) [33]. The work clearly indicates the reduction behaviour of Co(III)–glycine to Co(II)–glycine by ascorbic acid (in our work, it must be malonic acid from DES 2) [33].

The best extraction results were observed for the DES 2 with malonic acid. This result may be interpreted by the different acidity of the HBD—hydrogen bond donor—of the choline chloride–DESs. The acid strength of malonic acid (pK_a1_ = 2.83) is much higher than that of other organic acids used: citric acid, pK_a1_ = 3.13, lactic acid, pK_a1_ = 3.86, succinic acid, pK_a1_ = 4.20 and glutaric acid, pK_a1_ = 4.34 [44].

#### 2.2.2. Extraction with Bi-Functional Ionic Liquids

Further extraction was achieved with three bi-functional ionic liquids. The results obtained with [N_10,10,1,1_][Cyanex272] are presented in Table 8. The addition of H_2_O_2_ shows the recovery of metals at the level of 15–34 wt%. Better results were obtained with the addition of TCCA only for Li, *E*_Li_ = 55 wt%. The results obtained with [N_10,10,1,1_][D2EHPA] are presented in Table 9. The addition of H_2_O_2_ shows the recovery of Co, Ni, Li, Cu and Mn at the level of 1–25 wt%. Slightly better results were obtained with the addition of TCCA. The best results were observed for Co, Li and Mn, of *E*_Co_ = 47 wt%, *E*_Li_ = 49 wt% and *E*_Mn_ = 41 wt%. The only high extraction efficiency was observed in the case of PHM for Li, *E*_Li_ = 47 wt%. The extraction efficiency using (glycine + H_2_O_2_) was 21–39 wt% for all metals. The results obtained with [P_6,6,6,14_][Cyanex272] are presented in Table 10. The addition of H_2_O_2_ at pH = 3 shows metal recovery at the level of 10–36 wt%. Better results were observed with the addition of TCCA, but only for Li, *E*_Li_ = 51 wt%, and for Mn, *E*_Mn_ = 46 wt%. Unfortunately, the extraction efficiencies obtained when using the bi-functional ILs with various additives were not as attractive as in the case of the mixture (DES 2 + 15 g of glycine + H_2_O_2_) after two-stage extraction.

**Figure 8:**
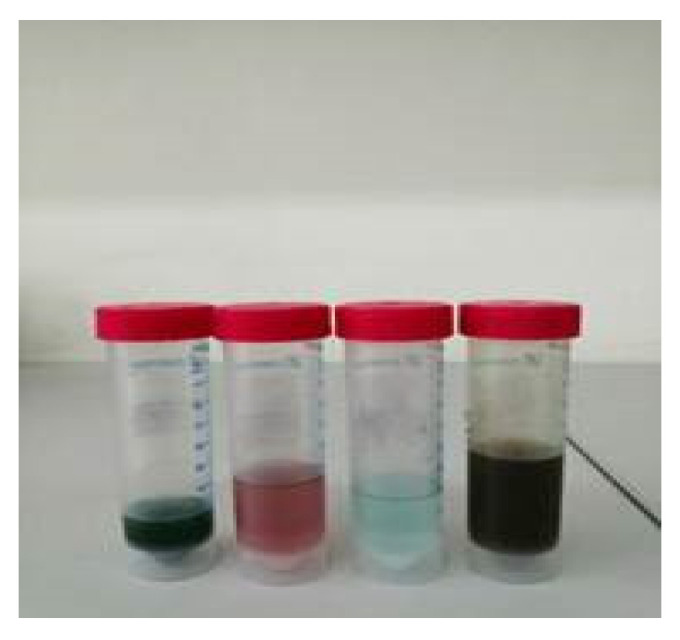
Products of extraction with bi-functional IL ([P_6,6,6,14_][Cyanex272] + TCCA): from left to right: (1) aqueous phase, dark green; (2) organic phase after the first stripping with 1.2 M H_2_SO_4_, pink; (3) solution after the second stripping with the 1.2 M H_2_SO_4_, blue; (4) organic phase (IL) after two stripping processes, brown.

The synthesis of ILs is presented in SMs. Figure 8 shows, as an example, extraction with bi-functional ILs ([P_6,6,6,14_][Cyanex272] + TCCA).

The synthesis of bi-functional ILs based on commercially available extractants such as Cyanex272, or D2EHPA with ammonium, or phosphonium IL has attracted interest due to the simplicity of synthesis and new properties. These ILs contain cations and anions with the functional groups which can act as both cationic and anionic extractants. In this work, new ILs were synthesized for metal extraction. The combination of these ions would increase the metal extraction. Cyanex 272, or D2EHPA, is widely known as a solvent used for the extraction of Co/Ni and other metal ions from the liquid phase. It is likely that the cations and anions of the synthetized ILs did not produce high selectivity for the extraction of metal ions compared to the [Cl]^−^ anion from the DES. 

The majority of the interpretations of the mechanism of these interactions are explained as “ion exchange” and/or “ion pairing” interactions for metal ions extracted from the aqueous (A) to the organic phase (O) [45]:

2 Me^+^_(A)_ + [Cl]^−^_(A)_ + [COO]^−^_(O)_ = MeCl_(A)_ + Me[COO]_(O)_ (2)

This reaction is possible after the extraction process of Me from the “black mass” into the liquid phase in the presence of IL or DES and the additives used in this work.

The same additives used with bi-functional ILs did not show high selectivity. The issue of separating metal ions from the obtained liquid phase needs to be solved in the next separation process. It is possible to find many studies and proposals in this field. Unfortunately, there is not usually a whole list of metals discussed in published works, such as Fe, Al, Cu, Mn, Li, Co and Ni. Our leachate solution after using a mixture of DES, water, DDACl, glycine and other additives is assumed to be an aqueous phase; therefore, the next extraction must be carried out with an organic solvent, for example D2EHPA, to separate some metals such as Fe, Cu and Al at different pHs with minimal extraction of Co and Ni. The last problem is the separation of Co and Ni which has been discussed in many papers over the last 20 years [46].

## 3. Materials and Methods

### 3.1. Analysis of the Solid LiPBs’ BM Material

The BM of LiPBs was provided by MB Recycling Sp. z o. o., a Waste Management Company in Kleszczów, Poland. The BM material (see Figure 1) was crushed and ground in a mortar into a powder of small diameter (approx. 1.2 mm). The qualitative elemental composition of the BM material was analyzed on a solid sample by SEM/EDS using a Jeol (Singapore) JSM-6490 LV scanning electron microscope (SEM) equipped with an energy-dispersive X-ray spectrometer (EDS). In the EDS analysis, the presence of lithium is not visible due to the limitations of the method (possibility to detect elements with an atomic mass equal to or greater than the atomic mass of boron). The presence of lithium was confirmed in quantitative analysis of the BM material. The Milestone (Fremont, CA, USA) UltraWAVE microwave digestion system was used in combination with the PerkinElmer (Waltham, MA, USA) AAnalyst 800 atomic absorption spectrometer (FAES technique for the determination of lithium and FAAS technique for other metals). Metals were determined after a microwave-assisted digestion of the sample in an UltraWAVE (Plattsburgh, NY, USA) mineralizer (using concentrated nitric acid as the solvent) followed by fusing the acid-insoluble matter with potassium pyrosulfate (and then dissolving the melted sample in dilute hydrochloric acid) to ensure complete sample dissolution prior to a quantitative analysis.

The results of the BM analysis for metal content are presented in Table 1. The EDS spectra, concerning only the micro-area of the BM surface, are shown in Figure 2 and Figures S1–S3 in the SMs.

### 3.2. Chemicals

Basic information (incl. chemical structure, name, abbreviation, molar mass and mass fraction purity) on the DESs, ILs ingredients and chemicals used in this work are listed in Table 11. The water used was deionized by a Millipore purification system. All other reagents employed in this work were of analytical grade.

The preparation of DESs, (1) {choline chloride + lactic acid, 1:2} [47], (2) {choline chloride + malonic acid, 1:1} [48], (3) {choline chloride + succinic acid, 1:1}, (4) {choline chloride + glutaric acid, 1:1}, (5,6) {choline chloride + citric acid, 1:1, and 2:1}, is described in the SMs. The choline chloride, [N_2OH,1,1,1_][Cl], used for the synthesis of DESs was dried under reduced pressure (10 hPa) at *T* = 323 K for 8 h. 

The bi-functional IL [P_6,6,6,14_][Cyanex272], CAS: 465527-59-7, was provided by IoLiTec. Two other ILs, [N_10,10,1,1_][Cyanex272] and [N_10,10,1,1_][D2EHPA], were synthesized for this work in our laboratory. The synthesis and NMR spectra recorded on the Bruker (Billerica, MA, USA) 300 MHz spectrometer in the presence of tetramethylsilane (TMS) as an internal standard are presented in the SMs. ILs used in the extraction were dried for 36 h at *T* = 340 K under reduced pressure, *p* = 6 kPa, and analyzed by Karl Fischer titration (Metrohm, Herisau, Switzerland, 716 DMS Titrino). The mass fraction of water in the samples was less than 760 × 10^−6^ g with an uncertainty of *u*(w.c.) = 10 × 10^−6^ g. The uncertainty of temperature measurements was ±0.1 K. All weighing was carried out using a Mettler Toledo (Greifensee, Switzerland) AB 204-S balance, with an accuracy of ±1 × 10^−4^ g. The Litmus bromothymol blue papers were used to measure the pH of the solution after extraction.

### 3.3. Extraction Procedure

A mixture of 15 g of the DES 1 was dissolved in 10 cm^3^ of water at *T* = 303 K and 10 g of BM, 8 cm^3^ of DDACl (50 wt% aqueous solution) was added in small portions, 5 cm^3^ of H_2_O_2_ (30 wt% aqueous solution) was added in small portions at *T* = 313 K and was stirred with a coated magnetic stirring bar under reflux for 2 h, 3000 rpm at *T* = 333 K at pH = 3 (regulated with 5 M H_2_SO_4_). Then, after sedimentation of the residual solid phase under the reduced pressure, the liquid phase was analyzed for the metal ions content. The solid to liquid (S/L) ratio was 10/38 g/cm^3^. The liquid phase was (32 cm^3^, 35.306 g). The lower, dark blue-greenish colour aqueous phase 30.5 cm^3^ + 1.5 cm^3^ of the upper organic phase was analyzed. The results are presented in Table 2. 

A mixture of 15 g of the DES 2 was dissolved in 10 cm^3^ of water at *T* = 303 K and 10 g of BM, 8 cm^3^ of DDACl (50 wt% aqueous solution) was added in small portions, 8 g of TCCA was dissolved in 22 cm^3^ of acetone added in small portions at *T* = 313 K and was stirred with a coated magnetic stirring bar under reflux for 2 h, 3000 rpm at *T* = 318 K at pH = 3 (regulated with 5M H_2_SO_4_). Then, after sedimentation of the residual solid phase under the reduced pressure, the liquid phase was analyzed for the metal ions content. The solid to liquid (S/L) ratio was 10/63 g/cm^3^. The lower, dark colour aqueous phase (30 cm^3^, 32,751 g) and the upper organic phase (12 cm^3^, 9841 g) were analyzed. The results are presented in Table 3. 

Similarly, mixtures of 10 g of BM and the remaining oxidizing additives were used (see the description of the mixtures in the SMs). The results are presented in Table 4, Table 5, Table 6 and Table 7. 

The mixture of DES 2 with glycine and H_2_O_2_ showed the best extraction efficiency results for Co, Ni and Li and was chosen for the two-stage extraction with the same recipe and different amounts of glycine (see Table S1 in the SMs and Figure 3). The effect of pH (pH = 3, 5, 7) on the two-stage extraction in the best system with 15 g of glycine at *T* = 333 K, for 2 h, is listed in Table S2 in the SMs and in Figure 5. The effect of temperature (*T* = 303 K, 318 K and 333 K) on the two-stage extraction with 15 g of glycine at pH = 3, for 2 h, using the best selected procedure, is shown in Table S3 and in Figure 6. The influence of the time of the two-stage extraction process (*t* = 30 min, 1 h, 1.5 h, 2 h) with 15 g of glycine at pH = 3, *T* = 333 K, on the extraction efficiency using the best selected procedure is shown in Table S4 and in Figure 7. The uncertainty of determining the extraction efficiency, taking into account the uncertainty of the results of determining the metal content in the starting BM material (plus material heterogeneity) and in the liquid phases after the extraction process (triple extraction test), was assumed to be 5%.

The further extraction was proposed with three bi-functional ILs: [N_10,10,1,1_][Cyanex272], [N_10,10,1,1_][D2EHPA] and [P_6,6,6,14_][Cyanex272]/toluene (diluent) with the addition of 8 cm^3^ of DDACl (50 wt% aqueous solution) and 5 cm^3^ of H_2_O_2_ (30 wt% aqueous solution), or 8 g of TCCA, 8 g of PHM, or (glycine, 8 g + H_2_O_2_, 5 cm^3^). The mixture was stirred with a coated magnetic stirring bar under reflux for 2 h, 3000 rpm, at *T* = 333 K at pH = 3 (regulated with 5M H_2_SO_4_). The aqueous to organic phase was A/O = 1/1. Two phases were obtained after the extraction and the organic phase was stripped with H_2_SO_4_ as is described in the SMs. The results are presented in Table 8, Table 9 and Table 10.

## 4. Conclusions

The article presents six DESs and three bi-functional ionic liquids with various additives used for the extraction of metals from the “black mass” of lithium-ion portable batteries (LiPBs). The aim was to recover Co, Ni, Li, Cu and Mn using different amounts of oxidizing additives or glutaric acid at different pHs and different extraction times. The preparation of six DESs containing choline chloride and acids, lactic acid (1:2, DES 1), malonic acid (1:1, DES 2), succinic acid (1:1, DES 3), glutaric acid (1:1, DES 4) and citric acid (1:1, DES 5), as well as citric acid (2:1, DES 6), is presented in addition to the synthesis of three bi-functional ILs: didecyldimethylammonium bis(2,4,4-trimethylpentyl) phosphinate, [N_10,10,1,1_][Cyanex272], didecyldimethylammonium bis(2-ethylhexyl) phosphate, [N_10,10,1,1_][D2EHPA] and trihexyltetradecylphosphonium bis(2,4,4-trimethylpentyl) phosphinate, [P_6,6,6,14_][Cyanex272]. Various additives, such as didecyldimethylammonium chloride, (DDACl) surfactant, hydrogen peroxide (H_2_O_2_), trichloroisocyanuric acid (TCCA), sodium dichloroisocyanurate (NaDCC), pentapotassium bis(peroxymonosulphate) bis(sulphate) (PHM) or (glycine + H_2_O_2_) or (glutaric acid + H_2_O_2_) were added to the DES or IL solvents for the extraction process at temperature *T* = 333 K for 2 h at pH = 3. DES 2 was chosen for the two-stage extraction with the addition of different amounts of glycine (8 g, 10 g, 15 g), at different pHs (pH = 3, 5, 7) and at different temperatures (303 K, 318 K, 333 K), as well as using different extraction times (0.5 h, 1 h, 1.5 h, 2 h) in order to search for the best extraction efficiencies. The highest efficiency of metal extraction was obtained with the mixture of {DES 2 + 15 g of glycine + H_2_O_2_} in the two-stage extraction at pH = 3, *T* = 333 K, and for 2 h. The addition of 15 g of glycine showed 100 wt% extraction efficiency for Co, Ni, Li and Mn and 75 wt% extraction efficiency for Cu. The extraction efficiency using bi-functional ILs for all metals was on the level of 35–50 wt%. This was demonstrated in a series of experiments at different pHs and temperatures. However, the issue of separating the metals from the final extraction solution has to be solved quickly, as rapidly increasing consumption of Li-ion batteries has increased the production over the past two years, resulting in a sharp increase in metal prices.

## Figures and Tables

**Table 1:** Metal content in the starting LiPBs’ BM material.

Co wt%	Ni wt%	Cu wt%	Mn wt%	Fe wt%	Al wt%	Zn wt%	Li wt%
2.95	8.6	4.0	3.4	1.9	1.65	0.067	2.2

**Table 2:** Results of metal extraction with DES 1 at *T* = 333 K, 2 h, extraction efficiency (*E*) at pH = 3.

Extrahent	Ion	g_0_ * (mg)	g_E_ * (mg)	*E* (wt%)
DES 1 + H_2_O_2_	Co(II)	295	82.44	28
	Ni(II)	860	246.36	29
	Li(I)	220	136.42	62
	Cu(II)	400	381.19	95
	Mn(II)	340	163.00	48
DES 1 + TCCA	Co(II)	295	141.44	48
	Ni(II)	860	492.10	57
	Li(I)	220	123.61	56
	Cu(II)	400	80.83	20
	Mn(II)	340	202.87	60
DES 1 + NaDCC × 2H_2_O	Co(II)	295	88.38	30
	Ni(II)	860	282.45	33
	Li(I)	220	95.39	43
	Cu(II)	400	111.04	28
	Mn(II)	340	100.37	30
DES 1 + PHM	Co(II)	295	108.61	37
	Ni(II)	860	132.32	15
	Li(I)	220	167.00	76
	Cu(II)	400	388.73	97
	Mn(II)	340	231.70	68
DES 1 + (glycine + H_2_O_2_)	Co(II)	295	153.75	52
	Ni(II)	860	452.75	53
	Li(I)	220	118.02	54
	Cu(II)	400	280.82	70
	Mn(II)	340	212.27	62
DES 1 + (glutaric acid + H_2_O_2_)	Co(II)	295	113.91	39
	Ni(II)	860	371.38	43
	Li(I)	220	103.76	47
	Cu(II)	400	329.28	82
	Mn(II)	340	165.28	49

* g_0_—metal content in the solid phase before extraction, g_E_—metal ion content in both aqueous phases after extraction [aqueous phase after extraction of the solid phase and aqueous phase after stripping the organic phase (TCCA only)].

**Table 3:** Results of metal extraction with DES 2 at *T* = 333 K, 2 h, extraction efficiency (*E*) at pH = 3.

Extrahent	Ion	g_0_ * (mg)	g_E_ * (mg)	*E* (wt%)
DES 2 + H_2_O_2_	Co(II)	295	40.65	14
	Ni(II)	860	104.36	12
	Li(I)	220	168.02	76
	Cu(II)	400	164.62	41
	Mn(II)	340	178.52	53
DES 2 + TCCA	Co(II)	295	175.07	59
	Ni(II)	860	429.03	50
	Li(I)	220	143.09	65
	Cu(II)	400	344.28	86
	Mn(II)	340	236.02	69
DES 2 + NaDCC × 2H_2_O	Co(II)	295	166.65	57
	Ni(II)	860	481.23	56
	Li(I)	220	144.06	66
	Cu(II)	400	146.64	37
	Mn(II)	340	210.80	62
DES 2 + PHM	Co(II)	295	58.60	20
	Ni(II)	860	19.55	2
	Li(I)	220	179.27	82
	Cu(II)	400	311.42	78
	Mn(II)	340	208.91	61
DES 2 + (glycine + H_2_O_2_)	Co(II)	295	177.22	60
	Ni(II)	860	568.86	66
	Li(I)	220	147.06	67
	Cu(II)	400	300.80	75
	Mn(II)	340	221.00	65
DES 2 + (glutaric acid + H_2_O_2_)	Co(II)	295	53.14	18
	Ni(II)	860	234.61	27
	Li(I)	220	157.42	72
	Cu(II)	400	82.98	21
	Mn(II)	340	152.34	45

* g_0_—metal content in the solid phase before extraction, g_E_—metal ion content in both aqueous phases after extraction [aqueous phase after extraction of the solid phase and aqueous phase after stripping the organic phase (TCCA only)].

**Table 4:** Results of metal extraction with DES 3 at *T* = 333 K, 2 h, extraction efficiency (*E*) at pH = 3.

Extrahent	Ion	g_0_ * (mg)	g_E_ * (mg)	*E* (wt%)
DES 3 + H_2_O_2_	Co(II)	295	103.37	35
	Ni(II)	860	284.53	33
	Li(I)	220	106.13	48
	Cu(II)	400	44.26	12
	Mn(II)	340	144.32	42
DES 3 + TCCA (*T* = 318 K)	Co(II)	295	157.82	53
	Ni(II)	860	381.36	44
	Li(I)	220	161.45	73
	Cu(II)	400	376.52	94
	Mn(II)	340	249.45	73
DES 3 + NaDCC × 2H_2_O	Co(II)	295	34.92	12
	Ni(II)	860	164.55	19
	Li(I)	220	131.92	60
	Cu(II)	400	191.64	48
	Mn(II)	340	28.21	8
DES 3 + PHM	Co(II)	295	60.03	20
	Ni(II)	860	13.87	2
	Li(I)	220	141.54	64
	Cu(II)	400	87.14	22
	Mn(II)	340	186.00	55
DES 3 + (glycine + H_2_O_2_)	Co(II)	295	116.19	39
	Ni(II)	860	378.38	44
	Li(I)	220	118.93	54
	Cu(II)	400	147.84	37
	Mn(II)	340	153.19	45
DES 3 + (glutaric acid + H_2_O_2_)	Co(II)	295	120.76	41
	Ni(II)	860	418.61	49
	Li(I)	220	115.26	52
	Cu(II)	400	109.57	27
	Mn(II)	340	146.68	43

* g_0_—metal content in the solid phase before extraction, g_E_—metal ion content in both aqueous phases after extraction [aqueous phase after extraction of the solid phase and aqueous phase after stripping the organic phase (TCCA only)].

**Table 5:** Results of metal extraction with DES 4 at *T* = 333 K, 2 h, extraction efficiency (*E*) at pH = 3.

Extrahent	Ion	g_0_ * (mg)	g_E_ * (mg)	*E* (wt%)
DES 4 + H_2_O_2_	Co(II)	295	133.41	45
	Ni(II)	860	360.16	42
	Li(I)	220	108.70	49
	Cu(II)	400	66.99	17
	Mn(II)	340	178.68	53
DES 4 + TCCA (*T* = 318 K)	Co(II)	295	151.76	51
	Ni(II)	860	426.40	50
	Li(I)	220	131.68	60
	Cu(II)	400	348.66	87
	Mn(II)	340	221.27	65
DES 4 + NaDCC × 2H_2_O	Co(II)	295	36.56	12
	Ni(II)	860	174.50	20
	Li(I)	220	112.19	51
	Cu(II)	400	130.56	33
	Mn(II)	340	36.31	11
DES 4 + PHM	Co(II)	295	55.46	19
	Ni(II)	860	12.67	2
	Li(I)	220	143.40	65
	Cu(II)	400	108.95	27
	Mn(II)	340	206.00	61
DES 4 + (glycine + H_2_O_2_)	Co(II)	295	109.32	37
	Ni(II)	860	333.66	39
	Li(I)	220	108.69	49
	Cu(II)	400	303.02	76
	Mn(II)	340	164.63	48
DES 4 + (glutaric acid + H_2_O_2_)	Co(II)	295	122.85	42
	Ni(II)	860	371.96	43
	Li(I)	220	106.67	49
	Cu(II)	400	81.87	20
	Mn(II)	340	167.52	49

* g_0_—metal content in the solid phase before extraction, g_E_—metal ion content in both aqueous phases after extraction [aqueous phase after extraction of the solid phase and aqueous phase after stripping the organic phase (TCCA only)].

**Table 6:** Results of metal extraction with DES 5 (1:1) at *T* = 333 K, 2 h, extraction efficiency (*E*) at pH = 3.

Extrahent	Ion	g_0_ * (mg)	g_E_ * (mg)	*E* (wt%)
DES 5 + H_2_O_2_	Co(II)	295	147.47	50
	Ni(II)	860	439.56	51
	Li(I)	220	136.47	62
	Cu(II)	400	335.74	84
	Mn(II)	340	206.09	61
DES 5 + TCCA (*T* = 318 K)	Co(II)	295	147.12	50
	Ni(II)	860	381.17	44
	Li(I)	220	132.92	60
	Cu(II)	400	350.78	88
	Mn(II)	340	206.69	61
DES 5 + NaDCC × 2H_2_O	Co(II)	295	142.85	48
	Ni(II)	860	442.10	51
	Li(I)	220	145.17	66
	Cu(II)	400	374.00	94
	Mn(II)	340	210.90	62
DES 5 + PHM	Co(II)	295	115,16	39
	Ni(II)	860	208.16	24
	Li(I)	220	193.01	88
	Cu(II)	400	347.42	87
	Mn(II)	340	250.52	74
DES 5 + (glycine + H_2_O_2_)	Co(II)	295	125.91	43
	Ni(II)	860	388.85	45
	Li(I)	220	134.53	62
	Cu(II)	400	341.59	85
	Mn(II)	340	178.51	53
DES 5 + (glutaric acid + H_2_O_2_)	Co(II)	295	116.05	39
	Ni(II)	860	367.34	43
	Li(I)	220	116.92	53
	Cu(II)	400	277.37	69
	Mn(II)	340	165.34	49

* g_0_—metal content in the solid phase before extraction, g_E_—metal ion content in both aqueous phases after extraction [aqueous phase after extraction of the solid phase and aqueous phase after stripping the organic phase (TCCA only)].

**Table 7:** Results of metal extraction with DES 6 (2:1) at *T* = 333 K, 2 h, extraction efficiency (*E*) at pH = 3.

Extrahent	Ion	g_0_ * (mg)	g_E_ * (mg)	*E* (wt%)
DES 6 + H_2_O_2_	Co(II)	295	151.95	52
	Ni(II)	860	510.33	59
	Li(I)	220	111.67	51
	Cu(II)	400	271.66	68
	Mn(II)	340	210.02	62
DES 6 + TCCA (*T* = 318 K)	Co(II)	295	178.91	61
	Ni(II)	860	173.15	20
	Li(I)	220	132.22	60
	Cu(II)	400	298.25	75
	Mn(II)	340	327.5	96
DES 6 + NaDCC × 2H_2_O	Co(II)	295	103.75	35
	Ni(II)	860	98.36	11
	Li(I)	220	92.80	42
	Cu(II)	400	259.72	65
	Mn(II)	340	229.67	68
DES 6 + PHM	Co(II)	295	94.19	32
	Ni(II)	860	311.5	36
	Li(I)	220	137.06	62
	Cu(II)	400	263.81	66
	Mn(II)	340	281.11	83
DES 6 + (glycine + H_2_O_2_)	Co(II)	295	168.76	57
	Ni(II)	860	169.91	20
	Li(I)	220	114.94	52
	Cu(II)	400	282.63	71
	Mn(II)	340	286.07	84
DES 6 + (glutaric acid + H_2_O_2_)	Co(II)	295	147.55	50
	Ni(II)	860	487.22	57
	Li(I)	220	108.68	49
	Cu(II)	400	272.95	68
	Mn(II)	340	271.84	80

* g_0_—metal content in the solid phase before extraction, g_E_—metal ion content in both aqueous phases after extraction [aqueous phase after extraction of the solid phase and aqueous phase after stripping the organic phase (TCCA only)].

**Table 8:** Results of metal extraction with [N_10,10,1,1_][Cyanex272] at *T* = 303 K, 2 h, extraction efficiency (*E*) at pH = 3.

Extrahent	Ion	g_0_ * (mg)	g_EAq_ * (mg)	g_EOrg_ * (mg)	*E* (wt%)
[N_10,10,1,1_][Cyanex272] + H_2_O_2_	Co(II)	295	14.15	45.99	20
	Ni(II)	860	50.74	75.32	15
	Li(I)	220	33.37	32.63	30
	Cu(II)	400	1.32	133.60	34
	Mn(II)	340	10.79	74.10	25
[N_10,10,1,1_][Cyanex272] + TCCA	Co(II)	295	12.62	69.93	28
	Ni(II)	860	99.03	141.69	28
	Li(I)	220	46.23	75.38	55
	Cu(II)	400	0.74	134.34	34
	Mn(II)	340	15.49	124.67	41
[N_10,10,1,1_][Cyanex272] + PHM	Co(II)	295	34.60	18.06	18
	Ni(II)	860	26.07	16.24	5
	Li(I)	220	80.33	20.82	46
	Cu(II)	400	7.25	106.76	29
	Mn(II)	340	26.07	15.75	12
[N_10,10,1,1_][Cyanex272] + (glycine + H_2_O_2_)	Co(II)	295	73.06	14.77	30
	Ni(II)	860	171.00	31.56	24
	Li(I)	220	66.57	14.87	37
	Cu(II)	400	114.62	45.77	40
	Mn(II)	340	122.86	131.56	75

* g_0_—metal content in the solid phase before extraction, g_E_—metal ion content in the aqueous or organic phase after the stripping process.

**Table 9:** Results of metal extraction with [N_10,10,1,1_][D2EHPA] at *T* = 303 K, 2 h, extraction efficiency (*E*) at pH = 3.

Extrahent	Ion	g_0_ * (mg)	g_EAq_ * (mg)	g_EOrg_ * (mg)	*E* (wt%)
[N_10,10,1,1_][D2EHPA] + H_2_O_2_	Co(II)	295	1.47	5.79	3
	Ni(II)	860	1.55	10.48	1
	Li(I)	220	21.61	15.37	17
	Cu(II)	400	2.31	97.83	25
	Mn(II)	340	3.02	12.61	5
[N_10,10,1,1_][D2EHPA] + TCCA	Co(II)	295	20.23	117.74	47
	Ni(II)	860	82.59	112.63	23
	Li(I)	220	57.03	51.27	49
	Cu(II)	400	1.63	119.37	30
	Mn(II)	340	39.82	100.58	41
[N_10,10,1,1_][D2EHPA] + PHM	Co(II)	295	35.87	6.12	14
	Ni(II)	860	19.22	6.01	3
	Li(I)	220	91.74	11.09	47
	Cu(II)	400	63.62	55.32	30
	Mn(II)	340	100.73	12.92	33
[N_10,10,1,1_][D2EHPA] + (glycine + H_2_O_2_)	Co(II)	295	73.97	6.49	27
	Ni(II)	860	168.63	12.63	21
	Li(I)	220	62.74	8.95	33
	Cu(II)	400	126.44	25.58	38
	Mn(II)	340	123.22	9.26	39

* g_0_—metal content in the solid phase before extraction, g_E_—metal ion content in the aqueous or organic phase after the stripping process.

**Table 10:** Results of metal extraction with [P_6,6,6,14_][Cyanex272] at *T* = 303 K, 2 h, extraction efficiency (*E*) at pH = 3.

Extrahent	Ion	g_0_ * (mg)	g_EAq_ * (mg)	g_EOrg_ * (mg)	*E* (wt%)
[P_6,6,6,14_][Cyanex272] + H_2_O_2_	Co(II)	295	41.89	34.69	26
	Ni(II)	860	115.04	43.50	18
	Li(I)	220	65.67	14.88	37
	Cu(II)	400	1.81	39.59	10
	Mn(II)	340	36.13	63.85	29
[P_6,6,6,14_][Cyanex272] + TCCA	Co(II)	295	14.96	82.97	33
	Ni(II)	860	221.54	25.84	29
	Li(I)	220	96.37	14.71	50
	Cu(II)	400	0.27	76.36	19
	Mn(II)	340	33.98	120.69	45
[P_6,6,6,14_][Cyanex272] + PHM	Co(II)	295	46.51	1.54	16
	Ni(II)	860	22.31	2.25	3
	Li(I)	220	98.73	1.34	46
	Cu(II)	400	69.86	16.09	22
	Mn(II)	340	109.97	2.66	33
[P_6,6,6,14_][Cyanex272] + (glycine + H_2_O_2_)	Co(II)	295	106.34	1.79	37
	Ni(II)	860	257.16	3.02	30
	Li(I)	220	90.73	0.85	42
	Cu(II)	400	142.89	4.03	37
	Mn(II)	340	200.30	2.73	60

* g_0_—metal content in the solid phase before extraction, g_E_—metal ion content in the aqueous or organic phase after the stripping process.

**Table 11:** Basic data on the DESs, the ILs ingredients and the chemicals used: structure, name, name abbreviation, supplier, CAS number, molar mass (M), mass fraction purity.

Chemical Structure	Name, Abbreviation, Supplier, CAS Number	Molar Mass M (g mol^−1^)	Purity * in Mass Percent (%)
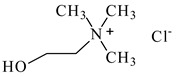	Choline chloride, [N_2OH,1,1,1_][Cl], Sigma-Aldrich, Darmstadt, Germany, CAS: 67-48-1	139.62	>98
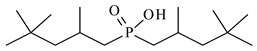	Bis(2,4,4-trimethylpentyl)phosphinic acid, Cyanex 272, Chem Scene LLC (Glenside, (PA)/ USA), CAS: 83411-71-6	290.42	90
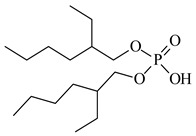	Bis(2-ethylhexyl) phosphate, D2EHPA, Heavy Water, Darmstadt, Germany, CAS: 298-07-7	322.40	>95
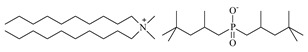	Didecyldimethylammonium bis(2,4,4-trimethylpentyl)phosphinate [N_10,10,1,1_][Cyanex272], Synthesized, Ł-IChP	616.12	>95
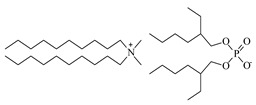	Didecyldimethylammonium bis(2-ethylhexyl)phosphate [N_10,10,1,1_][D2EHPA], C_38_H_82_NO_4_P, Synthesized, Ł-IChP	648.13	>95
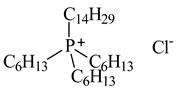	Trihexyltetradecylphosphonium chloride, Cyphos IL 101, [P_6,6,6,14_][Cl], IoLiTec, Heilbronn, Germany, CAS: 258864-54-9	519.42	>95
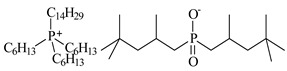	Trihexyltetradecylphosphonium bis(2,4,4-trimethylopentyl)phosphinate [P_6,6,6,14_][Cyanex272], ([P_6,6,6,14_][BTMPP]), IoLiTec, Heilbronn, Germany, CAS: 465527-59-7	773.27	>90
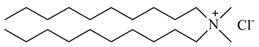	Didecyldimethylammonium chloride, [N_10,10,1,1_][Cl], DDACl, Alpinus Sp. z o.o. (Miszewko, Poland), CAS: 7173-51-5	362.16	50 wt% aq. solution
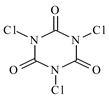	Trichloroizocyjanuric acid, C_3_Cl_3_N_3_O_3_, TCCA, Sigma-Aldrich, Darmstadt, Germany, CAS: 87-90-1,	232.40	95
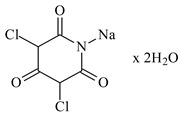	Sodium dichloroizocyjanurian dihydrat, C_3_Cl_2_N_3_NaO_2_ × 2H_2_O, NaDCC × 2H_2_O, Sigma-Aldrich, Darmstadt, Germany, CAS: 51580-86-0	239.99	≥98
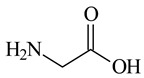	Glycine, C_2_H_5_NO_2_, Sigma-Aldrich, Darmstadt, Germany, CAS: 56-40-6	75.07	95
(2KHSO_5_·KHSO_4_·K_2_SO_4_)	Pentapotassium bis(peroxymonosulphate) bis(sulphate), PHM, Sigma-Aldrich, Darmstadt, Germany, CAS: 70693-62-8	614.76	98.0
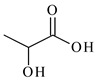	Lactic acid, C_3_H_6_O_3_, Sigma-Aldrich, Darmstadt, Germany, CAS: 50-21-5	90.08	98.0
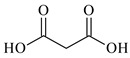	Malonic acid, C_3_H_4_O_4_, Reachim, Darmstadt, Germany, CAS: 141-82-2	104.06	99.0
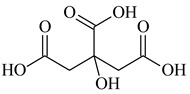	Citric Acid, C_6_H_8_O_7_, Riedel-de Haën, Seelze, Germany, CAS: 77-92-2	192.13	99.8
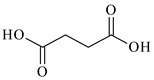	Succinic acid, C_4_H_6_O_4_, Avantor (POCh), Gliwice, Poland, CAS: 110-15-6	118.09	>99
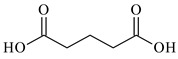	Glutaric acid, C_5_H_8_O_4_, Sigma-Aldrich, Darmstadt, Germany, CAS: 110-94-1	132.12	99
Kerosene	Kerosene, Dragon Poland Sp.z.o.o., Skawina, Poland	-	-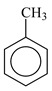	Toluene, C_6_H_5_CH_3_, Chempur, Karlsruhe, Germany, CAS: 108-88-3	92.14	98.8
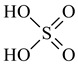	Sulphuric acid, H_2_SO_4_, Riedel-de Haën, Seelze, Germany, CAS 7664-93-9	98.08	96.0

* As stated by the supplier.

## Data Availability

The data presented in this study are available in the article and Supplementary Materials.

## Supplementary Materials

The following supporting information can be downloaded at: https://www.mdpi.com/article/10.3390/molecules29133142/s1, Figure S1: SEM image of the solid LiPBs BM sample and EDS spectrum of the micro-area marked in the image; Figure S2: SEM image of the solid LiPBs BM sample and EDS spectrum of the micro-area marked in the image; Figure S3: SEM image of the solid LiPBs BM sample and EDS spectrum of the micro-area marked in the image; Table S1: Results of metal extraction with DES 2 (1:1) at *T* = 333 K, 2 h, with different amounts of glycine, extraction efficiency (*E*) at pH = 3; Table S2: Results of metal extraction with {DES 2 (1:1) + 15 g of glycine + H_2_O_2_) at different pH at *T* = 333 K, 2 h, extraction efficiency (*E*); Table S3: Results of two-stage metal extraction with {DES 2 (1:1) + 15 g of glycine + H_2_O_2_) at different temperatures at pH = 3, 2 h, extraction efficiency (*E*); Table S4: Results of two-stage metal extraction with {DES 2 (1:1) + 15 g of glycine + H_2_O_2_) at different extraction times at pH = 3, *T* = 333 K, extraction efficiency (*E*). Refs. [24,47,48,49] are cited in Supplementary Materials.
