# Supplementary material for: Recovery of Metals from the “Black Mass” of Waste Portable Li-Ion Batteries with Choline Chloride-Based Deep Eutectic Solvents and Bi-Functional Ionic Liquids by Solvent Extraction
Source: Molecules. 2024 Jul 2;29(13):3142. doi: 10.3390/molecules29133142 (PMC11243434; doi:10.3390/molecules29133142)
Supplement: Supplementary file 1 [file molecules-29-03142-s001.zip › molecules-3028754-supplementary.pdf]

Molecules

Supplementary Material

Recovery of metals from the "black mass" of waste portable Li-ion batteries with choline chloride-based DESs and bi-functional ionic liquids by solvent extraction

Urszula Domańska[a*], Anna Wiśniewska[a], Zbigniew Dąbrowski[a], Dorota Kolasa[a], Kamil Wróbel[a], Jakub Lach[a]

[a]*ŁUKASIEWICZ Research Network – Industrial Chemistry Institute, Rydygiera 8, 01-793 Warsaw, Poland.*

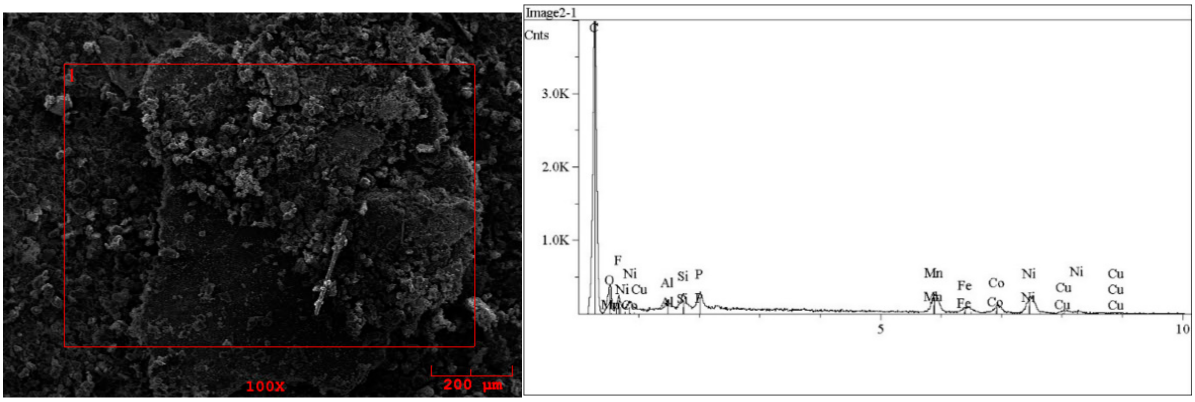

Figure S1. SEM image of the solid LiPBs BM sample and EDS spectrum of the micro-area marked in the image.

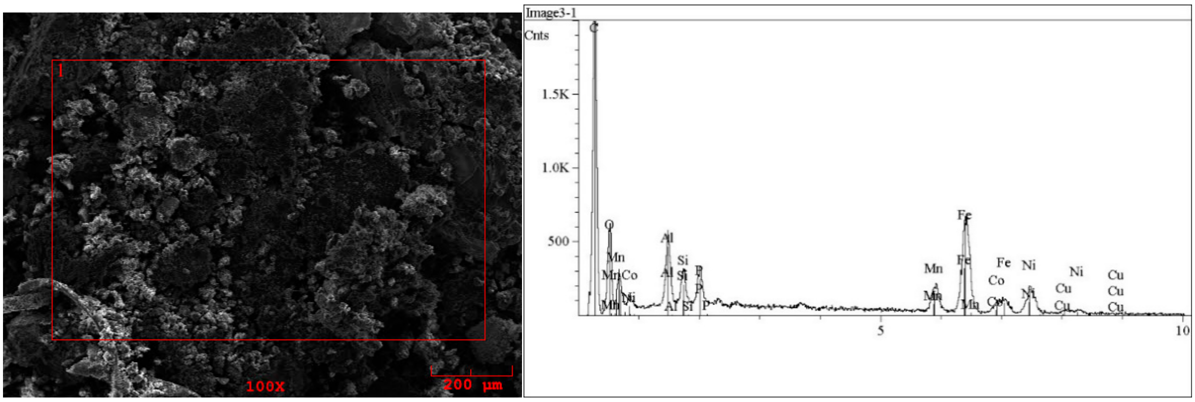

Figure S2. SEM image of the solid LiPBs BM sample and EDS spectrum of the micro-area marked in the image.

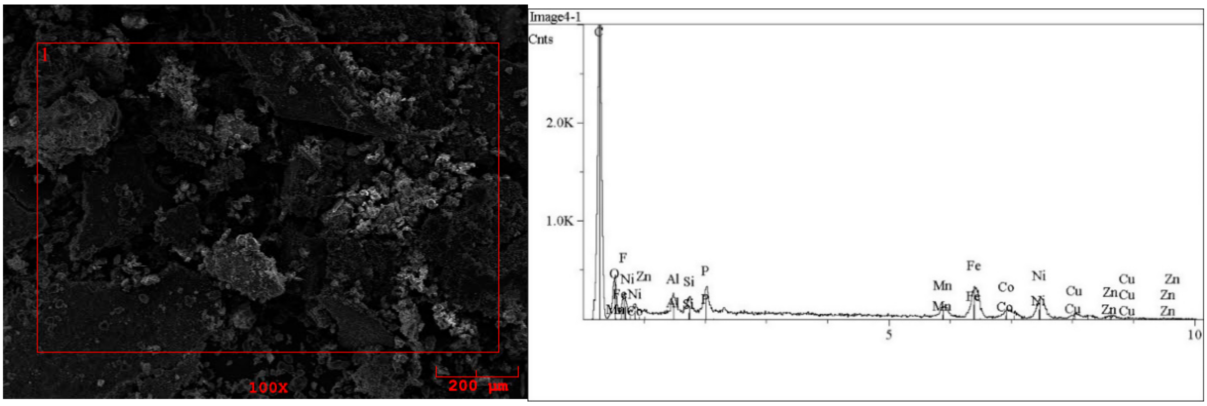

Figure S3. SEM image of the solid LiPBs BM sample and EDS spectrum of the micro-area marked in the image.

1. Preparation of DESs

Preparation of DESs is presented: {(1) choline chloride + lactic acid, 1:2} [47,49], {(2) choline chloride + malonic acid, 1:1} [48,49], {(3) choline chloride + succinic acid, 1:1}, {(4) choline chloride + glutaric acid, 1:1}, {(5)

choline chloride + citric acid, 1:1}. The choline chloride, $[N_{2OH,1,1,1}][Cl]$ used for the synthesis of DESs was dried under reduced pressure (10 hPa) at T = 323 K for 8 hours.

DES 1 (Choline chloride, $[N_{2OH,1,1,1}][Cl]$: lactic acid, 1:2): 43.55 g (0.31 mol) of $[N_{2OH,1,1,1}][Cl]$ (after drying for 8 h at T = 323 K under low pressure) was placed in a three-necked flask equipped with a reflux condenser. Then 56.13 g (0.62 mol) of lactic acid were added dropwise under an argon atmosphere. The mixture was stirred at T = 333 K for 8 h. After cooling to T = 323 K, the mixture was dried for 8 h under low pressure p = 20 hPa. The product obtained was a transparent liquid soluble in water.

DES 2 (Choline chloride, $[N_{2OH,1,1,1}][Cl]$: malonic acid, 1:1 [2,3]): 24.94 g (0.18 mol) of $[N_{2OH,1,1,1}][Cl]$ (after drying for 8 h at T = 323 K under low pressure) was placed in a three-necked flask equipped with a reflux condenser. Then 18.59 g (0.18 mol) of malonic acid were added in small portions. The mixture was stirred at T = 333 K for 8 h. After cooling to T = 323 K, the mixture was dried for 8 h at reduced pressure p = 20 hPa. The product obtained was a transparent liquid soluble in water.

DES 3 (Choline chloride, $[N_{2OH,1,1,1}][Cl]$: succinic acid, 1:1): 97.73 g (0.70 mol) of $[N_{2OH,1,1,1}][Cl]$ (after drying for 8 h at T = 323 K under low pressure) was placed in a three-necked flask equipped with a reflux condenser. Then 82.66 g (0.70 mol) of succinic acid were added in small portions under an argon atmosphere. The mixture was melted at T = 340 K. The mixture was stirred at T = 360 K for 9 h. After cooling to T = 323 K, the mixture was dried for 8 h under reduced pressure p = 20 hPa. The obtained product weighing 175.9 g was a yellow wax.

DES 4 (Choline chloride, $[N_{2OH,1,1,1}][Cl]$: glutaric acid, 1:1): 49.7 g (0.36 mol) of $[N_{2OH,1,1,1}][Cl]$ (after drying for 8 h at T = 323 K under low pressure) was placed in a three-necked flask equipped with a reflux condenser. Then 47.0 g (0.36 mol) of glutaric acid were added in small portions under an argon atmosphere. The mixture was stirred at T = 353 K for 9 h. After cooling to T = 323 K, the mixture was dried for 8 h under reduced pressure p = 20 hPa. The obtained product weighing 90.0 g was a honey colour liquid.

DES 5 (Choline chloride, $[N_{2OH,1,1,1}][Cl]$: citric acid, 1:1): 41.89 g (0.30 mol) of $[N_{2OH,1,1,1}][Cl]$ (after drying for 8 h at T = 323 K under reduced pressure) was placed in a three-necked flask equipped with a reflux condenser. Then 57.64 g (0.30 mol) of citric acid were added in small portions under an argon atmosphere. The mixture was stirred at T = 365 K for 9 h. After cooling to T = 323 K, the mixture was dried for 8 h under reduced pressure p = 20 hPa. The obtained product weighing 89.0 g was a yellow liquid at high temperature, and at a temperature bellow T = 353 K it transformed into the glassy phase.

DES 6 (Choline chloride, $[N_{2OH,1,1,1}][Cl]$: citric acid, 2:1): choline chloride was dried under reduced pressure of 2 mm Hg for 8 h (T = 333 K). In a 250 cm^3 three-neck flask equipped with a thermometer and a cooler condenser

83.77 g (0.60 mol) of choline chloride were placed. Then 57.64 g (0.30 mol) of citric acid were added under an argon atmosphere. The mixture was stirred magnetically at T = 377 K for 8 h. DES was obtained in the form of a clear, viscous liquid that undergoes a vitrification at a temperature below T = 323 K.

2. Synthesis of ILs

2.1. Trihexyltetradecylphosphonium bis(2,4,4-trimethylpentyl)phosphinate, $[P_{6,6,6,14}]$[Cyanex272], ($[P_{6,6,6,14}]$[BTMPP]), M = 773.27 g/mol; IoLiTec, CAS: 465527-59-7

2.2. Didecyldimethylammonium bis(2,4,4-trimethylpentyl)phosphinate, $[N_{10,10,1,1}]$[Cyanex272], $C_{38}H_{82}NO_2P$, M = 616.12 [4]

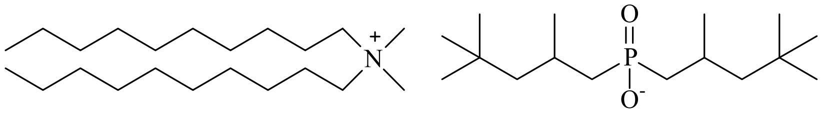

Didecyldimethylammonium bis(2,4,4-trimethylpentyl)phosphinate, $[N_{10,10,1,1}]$[Cyanex272] was obtained in a two-step reaction. In the first step, potassium bis(2,4,4-trimethylpentyl)phosphinate is obtained in the reaction of bis(2,4,4-trimethylpentyl)phosphinic acid (Cyanex272) with potassium hydroxide. Then didecyldimethylammonium chloride, $[N_{10,10,1,1}]$[Cl] reacts with potassium bis(2,4,4-trimethylpentyl)phosphinate to give didecyldimethylammonium bis(2,4,4-trimethylpentyl)phosphinate, $[N_{10,10,1,1}]$[Cyanex272]. Potassium chloride is a side product. The reaction is as follows:

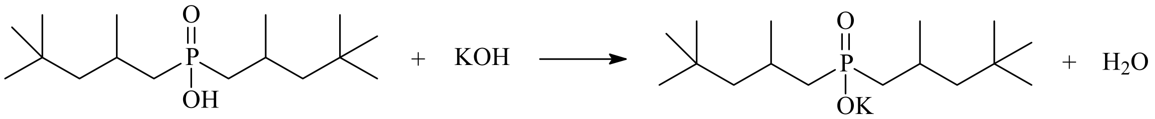

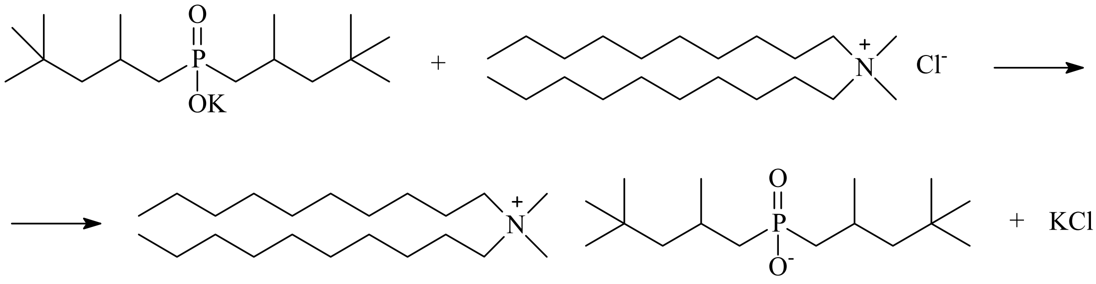

To a 250 cm^3 round bottom flask 16.13 g (0.05 mol) of Cyanex272, 90% and 2.80 g (0.05 mol) of potassium hydroxide dissolved in 30 cm^3 of water were added. The mixture was stirred magnetically for 3 h at T = 333–339 K. Then 18.11 g (0.05 mol) of [$N_{10,10,1,1}$][Cl] were added (as 50% aqueous solution ARQUAD 2.10-50, 36.22 g). Then all ingredients were stirred magnetically at T = 333–339 K for 6 h. 60 cm^3 of dichloromethane were added to the flask at T = 300 K and the two phases were separated. To the organic phase with the IL 30 cm^3 of water was added twice to remove KCl, the solvent was removed by distillation under reduced pressure and the residue was dried at a pressure of 1.3 hPa (T = 323 K, 10 h). 31.68 g (0.0495 mol) of didecyldimethylammonium bis(2,4,4-trimethylpentyl)phosphinate, [$N_{10,10,1,1}$][Cyanex272] were obtained. Yield 99.0%.

NMR spectra were recorded on a 300 MHz spectrometer in the presence of tetramethylsilane (TMS).

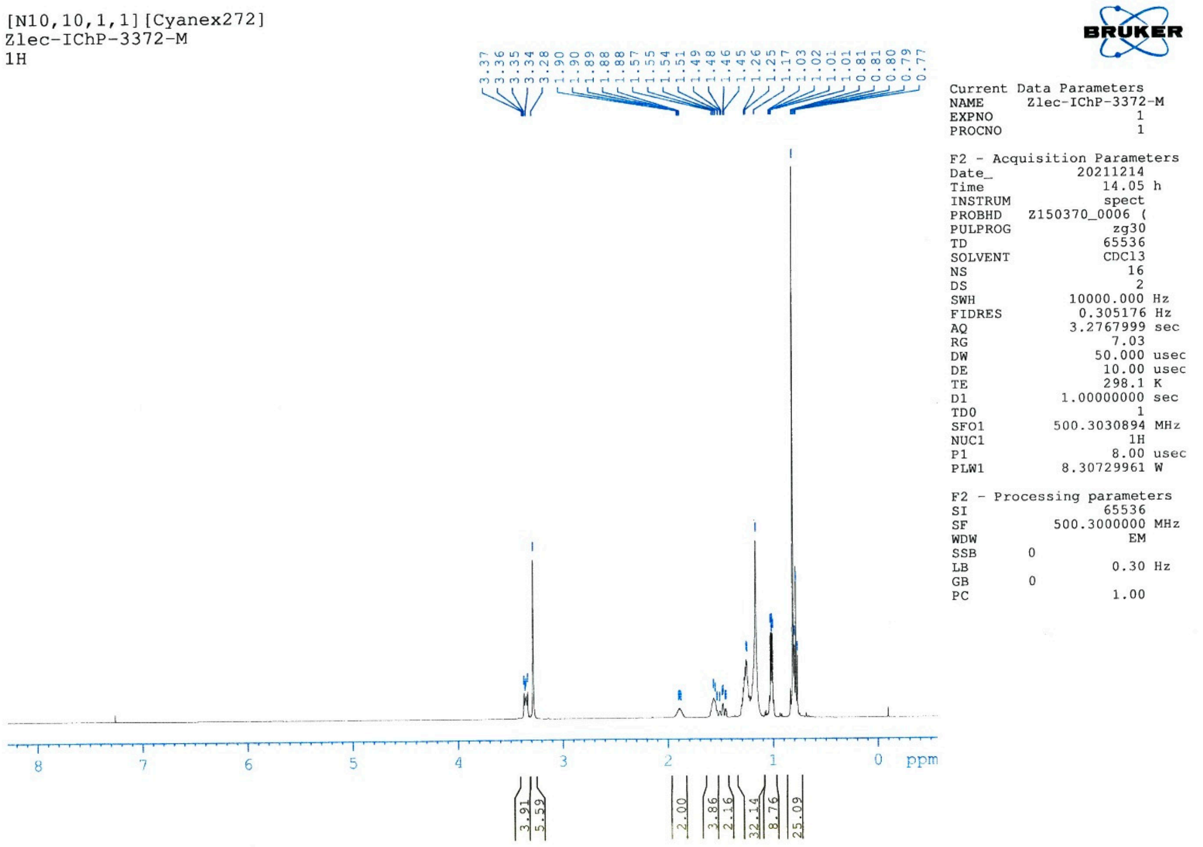

^{1}H NMR ($CDCl_3$, 500 MHz), δ: 0.77-0.84 (m, 24 H, 8 × CH_3), 1.00-1.04 (m, 8H), 1.13-1.30 (m, 32H, 16 × CH_2), 1.45-1.52 (m, 2H), 1.54-1.60 (m, 4H), 1.88-1.93 (m, 2H), 3.28 (s, 6H, 2 × $\mathbf{CH_3}$[N^+]), 3.34-3.37 m, 4H, 2 × $\mathbf{CH_2}$[N^+]) ppm.

^{13}C NMR ($CDCl_3$, 125.8 MHz), δ: 14.00 (2 × CH_3), 22.54 (2 × CH_2), 22.66 (2 × CH_2), 24.26 (CH_3), 24.29 (CH_3), 24.31 (CH_3), 24.34 (CH_3), 25.40 (d, CH, J = 3.1 Hz), 25.46 (d, CH, J = 3.1 Hz), 26.24 (CH_2), 29.14 (2 × CH_2), 29.15 (2 × CH_2), 29.29 (CH_2), 29.32 (2 × CH_2), 30.23 (2 × CH_3), 30.24 (2 × CH_3), 31.14 (2 × C), 31.73 (2 × CH_2), 42.06 (d, 2 × $\mathbf{CH_2}$[P=O]O^-), J = 86.2 Hz), 51.19 (2 × $\mathbf{CH_3}$[N^+]), 53.66 (CH_2), 53.73 (CH_2), 53.76 (CH_2), 53.83 (CH_2), 63.29 (2 × $\mathbf{CH_2}$[N^+]) ppm.

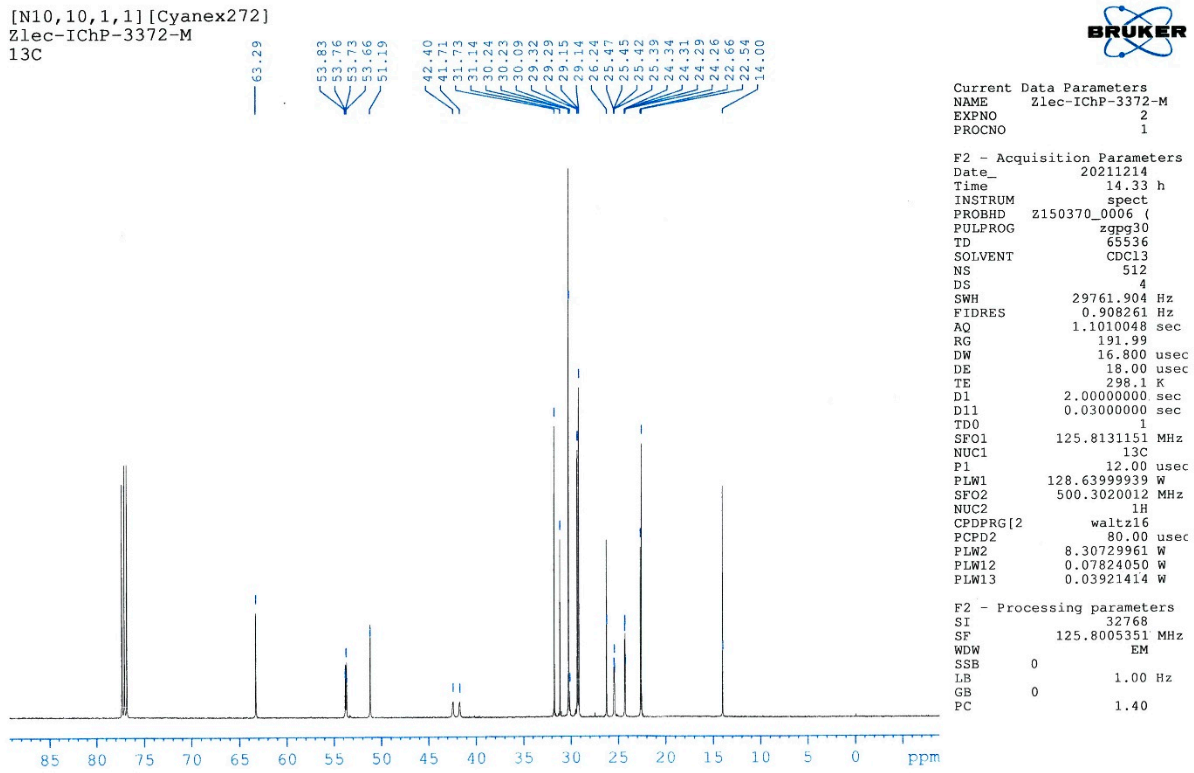

2.3. Didecyldimethylammonium bis(2-ethylhexyl)phosphate, [$N_{10,10,1,1}$][D2EHPA], $C_{38}H_{82}NO_4P$, M = 648,13 [4]

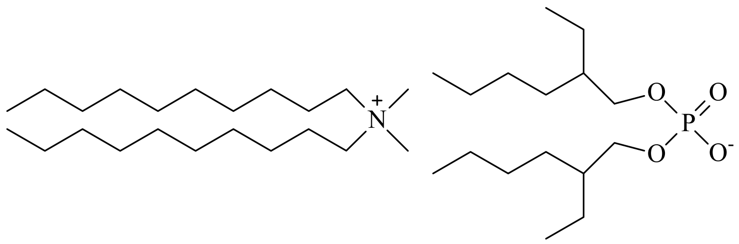

Didecyldimethylammonium bis(2-ethylhexyl)phosphate, [$N_{10,10,1,1}$][D2EHPA] was obtained in a two-steps reaction. In the first step, potassium bis(2-ethylhexyl)phosphate is obtained in the reaction of bis(2-

ethylhexyl)phosphoric acid with potassium hydroxide. Then didecyldimethylammonium chloride, [$N_{10,10,1,1}$][Cl] reacts with potassium bis(2-ethylhexyl)phosphate to give didecyldimethylammonium bis(2-ethylhexyl)phosphate, [$N_{10,10,1,1}$][D2EHPA]. Potassium chloride is a side product. The reaction is as follows:

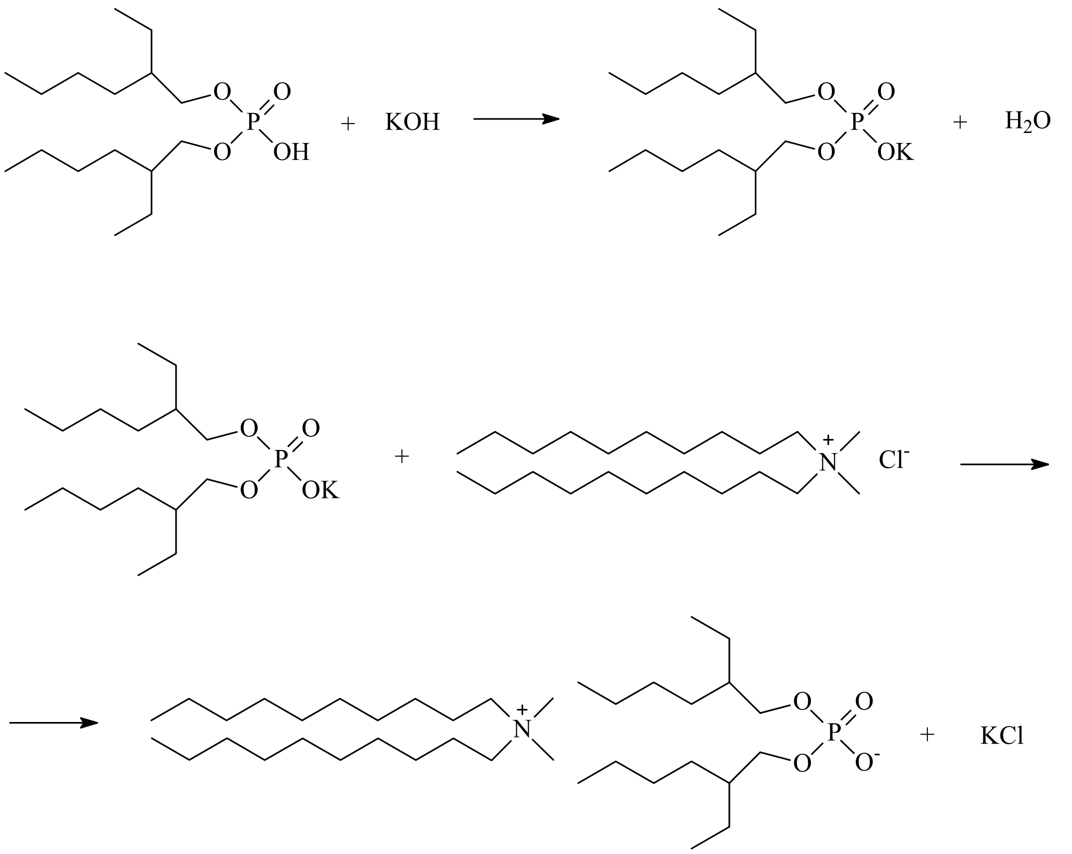

To a 500 cm^3 round bottom flask 32.24 g (0.1 mol) of D2EHPA and 5.61 g (0.1 mol) of potassium hydroxide dissolved in 50 cm^3 of water were added. The mixture was stirred magnetically for 2 h at T = 343 K. Then 36.22 g (0.1 mol) of [$N_{10,10,1,1}$][Cl] were added (the 50% aqueous solution ARQUAD 2.10-50 was used, 72.44 g). Then all ingredients were stirred magnetically at T = 333–339 K for 6 h. Then 60 cm^3 of dichloromethane were added to the flask at T = 300 K and the two phases were separated. To the organic phase with the IL 30 cm^3 of water was added twice to remove KCl, the solvent was removed by distillation under reduced pressure and the residue was dried at a pressure 1.3 hPa (T = 323 K, 10 h). 62.39 g (0.096 mol) of [$N_{10,10,1,1}$][D2EHPA] were obtained. Yield 96.0%.

NMR spectra were recorded on a 300 MHz spectrometer in the presence of tetramethylsilane (TMS).

^{1}H NMR ($CDCl_3$, 500 MHz), δ: 0.77-0.81 (m, 18H, 6 × CH_3), 1.13-1.28 (m, 42H), 1.33-1.39 (m, 2H), 1.41-1.46 (m, 2H), 1.56-1.62 (m, 4H, 2 × CH_2), 3.26 (s, 6H, 2 × **CH_3**[N^+]), 3.32-3.35 (m, 4H, 2 × **CH_2**[N^+]), 3.61-3.69 (m, 4H, 2 × **CH_2**O) ppm.

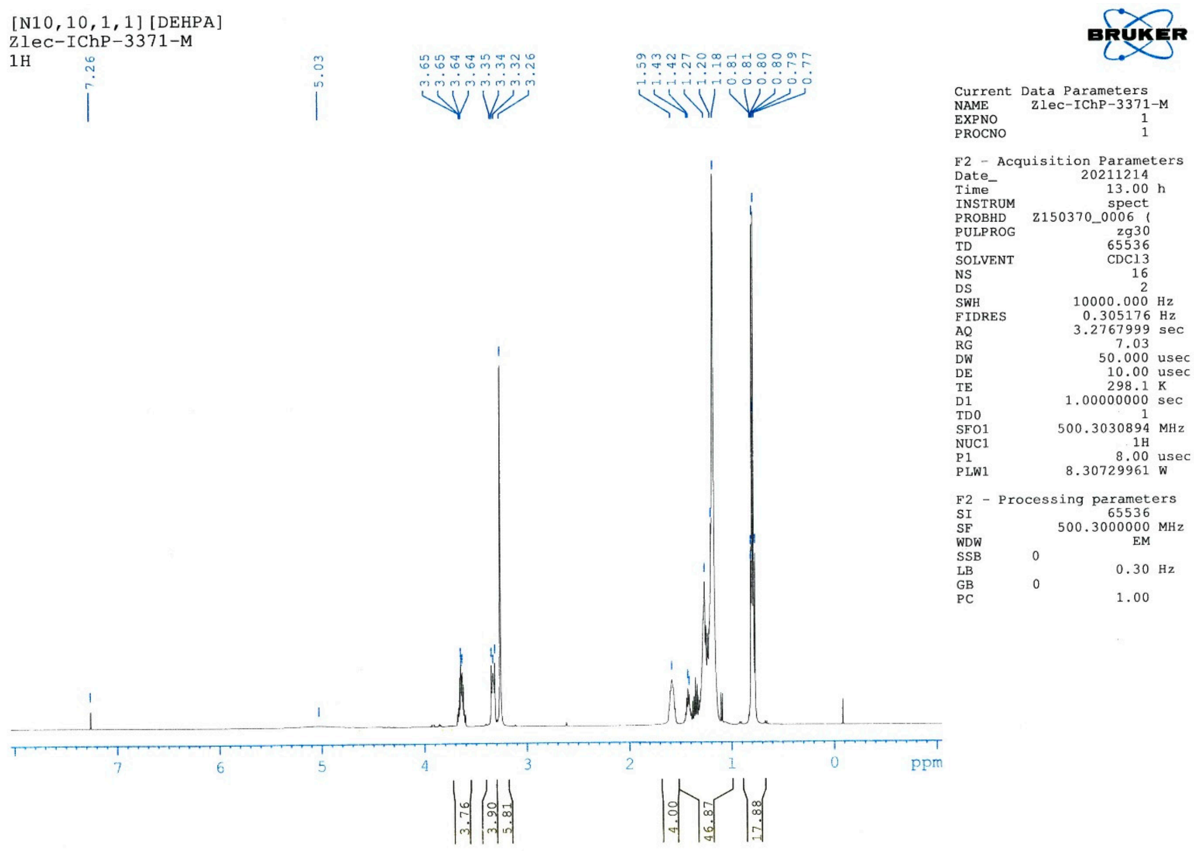

^{13}C NMR ($CDCl_3$, 125,8 MHz), δ: 10.83 (2 × CH_3),13.99 (2 × CH_3), 14.05 (2 × CH_3), 22.56 (2 × CH_2), 22.68 (2 × CH_2), 23.08 (2 × CH_2), 23.26 (2 × CH_2), 26.25 (2 × CH_2), 28.99 (2 × CH_2), 29.17 (2 × CH_2), 29.20 (2 × CH_2), 29.34 (2 × CH_2), 29.37 (2 × CH_2), 30.04 (2 × CH_2), 31.75 (2 × CH_2), 40.29 (CH), 40.35 (CH), 51.14 (2 × **CH_3**[N^+]), 63.21 (2 × **CH_2**[N^+]), 67.55 (CH_2O), 67.55 (CH_2O) ppm.

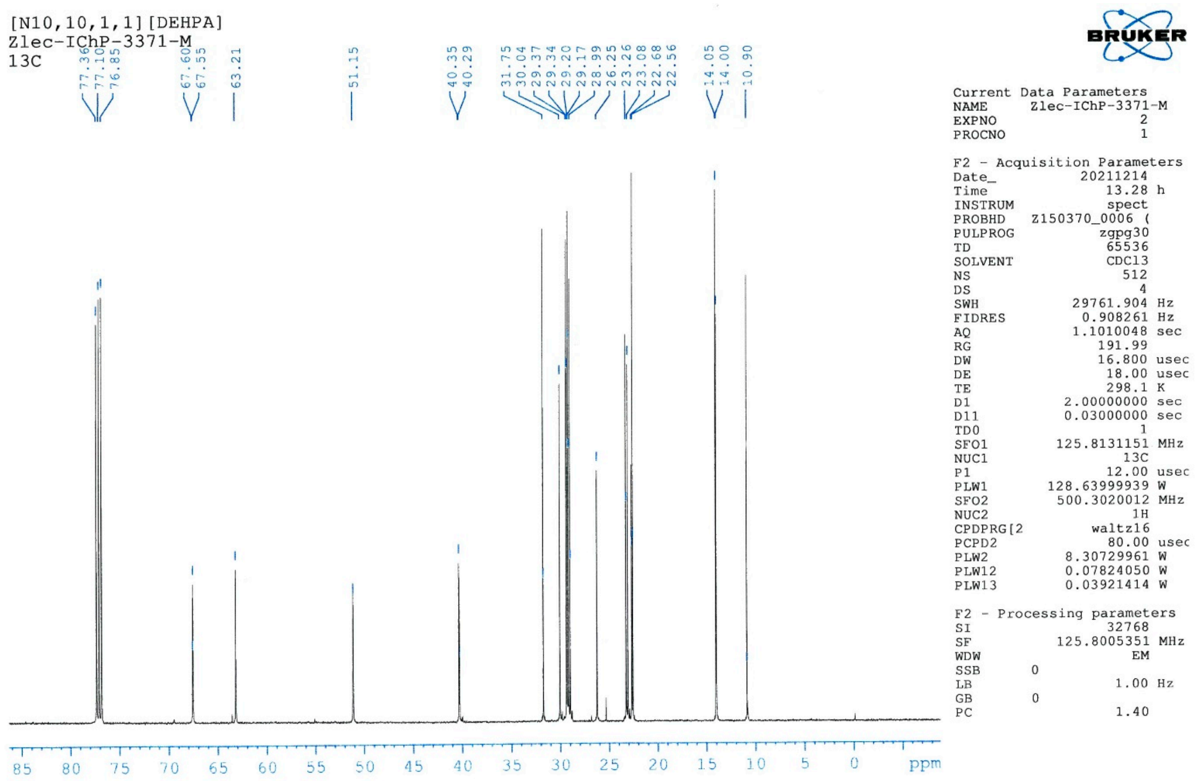

3. Extraction with DESs

A mixture of 15 g of the DES 1 dissolved in 10 cm^3 of water at T = 303 K, and 10 g of BM, 8 cm^3 of DDACl (50 wt% aqueous solution) in small portions, 8 g of **NaDCC x 2 H_2O** dissolved in 30 cm^3 of water in small portions at T = 313 K, was stirred with a coated magnetic stirring bar under reflux for 2 h, 3000 rpm at T = 333 K at pH = 3 (regulated with 5M H_2SO_4). Then, after sedimentation of the residual solid phase under reduced pressure, the liquid phase was analyzed for the metal ion content. The solid to liquid (S/L) ratio was 10/71 g/cm^3. The dark color aqueous phase (55 cm^3, 58.764 g) was analyzed.

A mixture of 15 g of the DES 1 dissolved in 10 cm^3 of water at T = 303 K, and 10 g of BM, 8 cm^3 of DDACl (50 wt% aqueous solution) in small portions, 8 g of **PHM** dissolved in 30 cm^3 of water in small portions at T = 313 K, was stirred with a coated magnetic stirring bar under reflux for 2 h, 3000 rpm at T = 333 K at pH = 3 (regulated with 5M H_2SO_4). Then, after sedimentation of the residual solid phase under reduced pressure, the liquid phase was analyzed for the metal ion content. The solid to liquid (S/L) ratio was 10/71 g/cm^3. The dark color aqueous phase (63 cm^3, 66.427 g) was analysed.

A mixture of 15 g of the DES 1 dissolved in 10 cm^3 of water at T = 303 K, and 10 g of BM, 8 cm^3 of DDACl (50 wt% aqueous solution) in small portions, 8 g of glycine dissolved in 15 cm^3 of water in small portions

and 5 cm^3 of **H_2O_2** (30 wt% aqueous solution) at T = 313 K, was stirred with a coated magnetic stirring bar under reflux for 2 h, 3000 rpm at T = 333 K at pH = 3 (regulated with 5M H_2SO_4). Then, after sedimentation of the residual solid phase under reduced pressure, the liquid phase was analyzed for the metal ion content. The solid to liquid (S/L) ratio was 10/61 g/cm^3. The lower dark blue aqueous phase and the second yellow-brown upper phase (2 cm^3) were analysed (50 cm^3, 56.985 g).

A mixture of 15 g of the DES 1 dissolved in 10 cm^3 of water at T = 303 K, and 10 g of BM, 8 cm^3 of DDACl (50 wt% aqueous solution) in small portions, 5 g of **glutaric acid** dissolved in 10 cm^3 of water in small portions and 5 cm^3 of **H_2O_2** (30 wt% aqueous solution) at T = 323 K, was stirred with a coated magnetic stirring bar under reflux for 2 h, 3000 rpm at T = 333 K at pH = 3 (regulated with 5M H_2SO_4). Then, after sedimentation of the residual solid phase under reduced pressure, the liquid phase was analyzed for the metal ion content. The solid to liquid (S/L) ratio was 10/61 g/cm^3. The lower blue-green aqueous phase and the second upper phase (2 cm^3) were analysed (45 cm^3, 49.059 g).

In general, all DESs were used with different oxidizing substances and glutaric acid as described above, unless otherwise stated.

A mixture of 15 g of the DES 2 dissolved in 10 cm^3 of water at T = 303 K, and 10 g of BM, 8 cm^3 of DDACl (50 wt% aqueous solution) in small portions, 8 g of **glycine** dissolved in 25 cm^3 of water in small portions and 5 cm3 of **H_2O_2** (30 wt% aqueous solution) at T = 313 K, was stirred with a coated magnetic stirring bar under reflux for 2 h, 3000 rpm at T = 333 K at pH = 3 (regulated with 5M H_2SO_4). Then, after sedimentation of the residual solid phase under reduced pressure, the solid phase was dried at 333 K for 5 h. The lower dark blue aqueous phase (60 cm^3, 65.67 g) and the yellow-brown upper phase (2 cm^3) were obtained. The rest of a solid phase (7 g) was extracted a second time. A mixture of 10.5 g of the DES 2 dissolved in 7 cm^3 of water at T = 303 K and 7 g of BM, 5.6 cm^3 of DDACl (50 wt% aqueous solution) in small portions, 5.6 g of **glycine** dissolved in 17.5 cm^3 of water in small portions and 3.5 cm^3 of **H_2O_2** (30 wt% aqueous solution) at T = 313 K, was stirred with a coated magnetic stirring bar under reflux for 2 h, 3000 rpm at T = 333 K at pH = 3 (regulated with 5M H_2SO_4). Then, after sedimentation of the residual solid phase under reduced pressure, the liquid phase from the first and the second stage of the extraction was analyzed for the metal ion content. The lower violet aqueous phase and small amount of the second brown upper phase (1.5 cm^3) were analysed (41 cm^3, 45.07 g).

4. Extraction with bi-functional ILs

4.1. Extraction with [$N_{10,10,1,1}$][Cyanex 272]

A mixture of 15 g of the IL dissolved in 7.5 cm^3 of naphtha plus 10 cm^3 of water at T = 303 K, and 10 g of BM, 8 cm^3 of DDACl (50 wt% aqueous solution), 5 cm^3 of H_2O_2 (30 wt% aqueous solution) added in small portions at T = 308 K, was stirred with a coated magnetic stirring bar under reflux for 2 h, 3000 rpm at T = 333 K at pH = 3 (regulated with 5M H_2SO_4). The O/A = 1/2. Then, after sedimentation of the residual solid phase under reduced pressure, the liquid phase was analyzed for the metal ion content. The lower aqueous phase (5.5 cm^3, 5.89 g) and the upper dark blue organic phase (32 cm^3) were analyzed after stripping.

Stripping 1. To 32 cm^3 of the organic phase 16 cm^3 of 1.2 M H_2SO_4 were added and stirred at temperature T = 323 K for 20 min. After 30 min two phases were obtained: 31.6 $cm^3$of the upper brown organic phase and 15.6 cm^3 (16.56 g) of the lower acidic aqueous phase.

Stripping 2. To 31.6 cm^3 of the organic phase 15.8 cm^3 of 1.2 M H_2SO_4 were added and stirred at temperature T = 323 K for 20 min. After 30 min two phases were obtained: 28.4 cm^3 (25.89g) of the upper light-brown organic phase and 17.26 cm^3 (17.93 g) of the lower acidic aqueous phase.

A mixture of 15 g of IL dissolved in in 7.5 cm^3 of naphtha plus 10 cm^3 of water at T = 303 K, and 10 g of BM, 8 cm^3 of DDACl (50 wt% aqueous solution) added in small portions, 8 g of TCCA dissolved in 22 cm^3 of acetone added in small portions at T = 313 K, were stirred with a coated magnetic stirring bar under reflux for 2 h, 3000 rpm at **T = 318 K** at pH = 3 (regulated with 5M H_2SO_4). Then, after sedimentation of the residual solid phase under reduced pressure, the liquid phases were analyzed for the metal ion content. The O/A = 1/2. The upper dark-blue color organic phase (46.8 cm^3) + 4.6 cm^3 (4.50 g) of the lower green-grey aqueous phase were obtained.

Stripping 1. To 46.8 cm^3 of the organic phase 23.4 cm^3 of 1.2 M H_2SO_4 were added and stirred at temperature T = 323 K for 20 min. After 30 min two phases were obtained: 36 $cm^3$of the upper brown organic phase and 31.2 cm^3 (31.09 g) of the lower acidic light-pink aqueous phase.

Stripping 2. To 36 cm^3 of the organic phase 18 cm^3 of 1.2 M H_2SO_4 were added and stirred at temperature T = 323 K for 20 min. After 30 min two phases were obtained: 31.6 cm^3 (31.09 g) of the upper orange-brown organic phase and 20.2 cm^3 (23.43 g) of the lower acidic green-blue aqueous phase.

A mixture of 15 g of IL dissolved in 7.5 cm^3 of naphtha plus 30 cm^3 of water at $T = 303$ K, and 10 g of BM, 8 cm^3 of DDACl (50 wt% aqueous solution) added in small portions, 8 g of PHM added in small portions at $T = 313$ K, was stirred with a coated magnetic stirring bar under reflux for 2 h, 3000 rpm at $T = 333$ K at pH = 3 (regulated with 5M H_2SO_4). Then, after sedimentation of the residual solid phase under reduced pressure, the liquid phases were analyzed for the metal ion content. The O/A = 1/2. The upper dark-blue color organic phase 30 cm^3 (27.82 g) + 20.5 cm^3 (23.31 g) of the lower pink aqueous phase were obtained.

Stripping 1. To 30 cm^3 of the organic phase 15 cm^3 of 1.2 M H_2SO_4 were added and stirred at temperature $T = 323$ K for 20 min. After 30 min two phases were obtained: 26 cm^3(20.94 g) of the upper brown organic phase and 16.8 cm^3 (18.14 g) of the lower blue aqueous phase.

Stripping 2. To 26 cm^3 of the organic phase 13 cm^3 of 1.2 M H_2SO_4 were added and stirred at temperature $T = 323$ K for 20 min. After 30 min two phases were obtained: 24.8 cm^3 (20.94 g) of the upper light-brown organic phase and 14 cm^3 (14.89 g) of the lower acidic blue aqueous phase.

A mixture of 15 g of IL dissolved in in 7.5 cm^3 of naphtha plus 10 cm^3 of water at $T = 303$ K, and 10 g of BM, 8 cm^3 of DDACl (50 wt% aqueous solution) added in small portions, 8 g of glycine in 20 cm^3 of water and 5 $cm^3$of H_2O_2 (30 wt% aqueous solution) added in small portions at $T = 313$ K, was stirred with a coated magnetic stirring bar for 2 h, 3000 rpm at $T = 333$ K at pH = 3 (regulated with 5M H_2SO_4). Then, after sedimentation of the residual solid phase under reduced pressure, the liquid phases were analysed for the metal ion content. The O/A = 1/2, 31.8 cm^3 (29.30 g) of the upper green-grey colour organic phase and 28.2 $cm^3$of the lower blue aqueous phase were obtained.

Stripping 1. To 31.8 cm^3 of the organic phase 16 cm^3 of 1.2 M H_2SO_4 were added and stirred at temperature $T = 323$ K for 20 min. After 30 min two phases were obtained: 28.9 cm^3 of the upper brown organic phase and 16.3 cm^3 (17.47 g) of the lower grey aqueous phase.

Stripping 2. To 28.9cm^3 of the organic phase 14.4 cm^3 of 1.2 M H_2SO_4 were added and stirred at temperature $T = 323$ K for 20 min. After 30 min two phases were obtained: 25.9 cm^3 (24.33 g) of the upper light-brown organic phase and 16.4 cm^3 (17.32 g) of the lower acidic yellow aqueous phase.

In general, all bi-functional ILs were used with different oxidizing substances as described above, unless otherwise stated.

References

[24] A. Łukomska, A. Wiśniewska, Z. Dąbrowski, J. Lach, K. Wróbel, D. Kolasa, U. Domańska, Recovery of Metals from Electronic Waste-Printed Circuit Boards by Ionic Liquids, DESs and Organophosphorous-Based Acid Extraction. Molecules 27 (2022) 4984, https://doi.org/10.3390/molecules27154984.

[47] W-H. Zhang, M-N. Chen, Y. Hao, X. Jiang, X-L. Zhou, Z-H. Zhang, Choline chloride and lactic acid: A natural deep eutectic solvent for one-pot rapid construction of spiro[indoline-3,4′-pyrazolo[3,4-*b*]pyridines], J. Mol. Liq. 278 (2019) 124–129, https://doi.org/10.1016/j.molliq.2019.01.065.

[48] A.P. Abbott, D. Boothby, G. Capper, D.L. Davies, R.K. Rasheed, Deep Eutectic Solvents Formed between Choline Chloride and Carboxylic Acids: Versatile Alternatives to Ionic Liquids, J. Am. Chem. Soc. 126 (2004) 9142–9147, https://doi.org/10.1021/ja048266j.

[49] A. Łukomska, A. Wiśniewska, Z. Dąbrowski, D. Kolasa, S. Luchcińska, J. Lach, K. Wróbel, U. Domańska, Recovery of zinc and manganese from "black mass" of waste Zn-MnO_2 alkaline batteries by solvent extraction technique with ionic liquids, DESs and organophosphorous-based acids. J. Mol. Liq. 338 (2021) 116590, https://doi.org/10.1016/j.molliq.2021.116590.

Table S1

Results of metal extraction with DES 2 (1:1) at T = 333 K, 2 h, with different amounts of glycine, extraction efficiency (E) at pH = 3.

Extractant	Ion	g_0* (mg)	g_E* (mg)	E (wt%)
DES 2 + **8 g** of glycine + H_2O_2	Co(II)	295	314.17	100**
	Ni(II)	860	769.39	90
	Li(I)	220	190.58	87
	Cu(II)	400	276.36	69
	Mn(II)	340	357.37	100**

DES 2<br><br>+ **10 g** of glycine + H_2O_2	Co(II)	295	314.20	100**
	Ni(II)	860	795.50	93
	Li(I)	220	198.00	90
	Cu(II)	400	286.00	72
	Mn(II)	340	357.30	100**
DES 2<br><br>+ **15 g** of glycine + H_2O_2	Co(II)	295	321.43	100**
	Ni(II)	860	873.96	100**
	Li(I)	220	221.55	100**
	Cu(II)	400	300.28	75
	Mn(II)	340	365.06	100**

* g_0 – metal content in the solid phase before the extraction, g_E – metal ion content in both aqueous phases after extraction (aqueous phase after extraction of solid phase, aqueous phase after stripping the organic phase).

**Extraction efficiency exceeding 100% (100.7-109%) is due to the uncertainty of the metal determination results and the heterogeneity of the solid material. The uncertainty of determining the extraction efficiency, taking into account the uncertainty of determining the metal content in the starting BM material (plus material heterogeneity) and after the extraction process (triple extraction test), was assumed to be 5%.

Table S2

Results of metal extraction with {DES 2 (1:1) + 15 g of glycine + H_2O_2) at different pH at T = 333 K, 2 h, extraction efficiency (E).

Extractant	Ion	g_0* (mg)	g_E* (mg)	E (wt%)	pH
DES 2	Co(II)	295	321.43	100**	3
	Ni(II)	860	873.96	100**	
	Li(I)	220	221.55	100**	
	Cu(II)	400	300.28	75	
	Mn(II)	340	365.06	100*	
DES 2	Co(II)	295	311.20	100**	5
	Ni(II)	860	863.00	100**	
	Li(I)	220	237.29	100**	
	Cu(II)	400	290.00	73	
	Mn(II)	340	365.43	100**	
DES 2	Co(II)	295	295.80	100**	7
	Ni(II)	860	842.59	98	
	Li(I)	220	238.23	100**	
	Cu(II)	400	311.65	77	
	Mn(II)	340	361.04	100**	

* g_0 – metal content in the solid phase before the extraction, g_E – metal ion content in both aqueous phases after extraction (aqueous phase after extraction of solid phase, aqueous phase after stripping the organic phase).

**Extraction efficiency exceeding 100% (100.3-109%) is due to the uncertainty of the metal determination results and the heterogeneity of the solid material. The uncertainty of determining the extraction efficiency, taking into account the uncertainty of determining the metal content in the starting BM material (plus material heterogeneity) and after the extraction process (triple extraction test), was assumed to be 5%.

Table S3

Results of two-stage metal extraction with {DES 2 (1:1) + 15 g of glycine + H_2O_2) at different temperatures at pH = 3, 2 h, extraction efficiency (*E*).

Extractant	Ion	g_0^* (mg)	g_E^* (mg)	E (wt%)
DES 2 (303 K)	Co(II)	295	238.76	81
	Ni(II)	860	474.44	55
	Li(I)	220	143.32	65
	Cu(II)	400	319.05	80
	Mn(II)	340	335.57	99
DES 2 (318 K)	Co(II)	295	287.62	98
	Ni(II)	860	660.48	77
	Li(I)	220	184.48	84
	Cu(II)	400	314.05	79
	Mn(II)	340	346.80	100**
DES 2 (333 K)	Co(II)	295	321.4	100**
	Ni(II)	860	874.0	100**

	Li(I)	220	221.5	100**
	Cu(II)	400	300.3	75
	Mn(II)	340	365.1	100**

* g_0 – metal content in the solid phase before the extraction, g_E – metal ion content in both aqueous phases after extraction (aqueous phase after extraction of solid phase, aqueous phase after stripping the organic phase).

**Extraction efficiency exceeding 100% (100.7-109%) is due to the uncertainty of the metal determination results and the heterogeneity of the solid material. The uncertainty of determining the extraction efficiency, taking into account the uncertainty of determining the metal content in the starting BM material (plus material heterogeneity) and after the extraction process (triple extraction test), was assumed to be 5%.

Table S4

Results of two-stage metal extraction with {DES 2 (1:1) + 15 g of glycine + H_2O_2) at different extraction times at pH = 3, T = 333 K, extraction efficiency (E).

Extractant	Ion	g_0* (mg)	g_E* (mg)	E (wt%)
DES 2 (0.5 h)	Co(II)	295	282.72	96
	Ni(II)	860	618.21	72
	Li(I)	220	165.15	75
	Cu(II)	400	311.20	78
	Mn(II)	340	355.30	100**
DES 2 (1 h)	Co(II)	295	293.80	100
	Ni(II)	860	638.51	74
	Li(I)	220	177.45	81

	Cu(II)	400	316.69	79
	Mn(II)	340	357.00	100**
DES 2 (1.5 h)	Co(II)	295	307.09	100**
	Ni(II)	860	678.68	79
	Li(I)	220	192.50	88
	Cu(II)	400	305.56	76
	Mn(II)	340	360.40	100**
DES 2 (2 h)	Co(II)	295	321.40	100**
	Ni(II)	860	874.00	100**
	Li(I)	220	221.50	100**
	Cu(II)	400	300.30	75
	Mn(II)	340	365.10	100**

* g_0 – metal content in the solid phase before the extraction, g_E – metal ion content in the both aqueous phases after extraction (aqueous phase after extraction of solid phase, aqueous phase after stripping the organic phase).

**Extraction efficiency exceeding 100% (104.5-107.4%) is due to the uncertainty of the metal determination results and the heterogeneity of the solid material. The uncertainty of determining the extraction efficiency, taking into account the uncertainty of determining the metal content in the starting BM material (plus material heterogeneity) and after the extraction process (triple extraction test), was assumed to be 5%.
